# The BCL-2 family protein BCL-RAMBO interacts and cooperates with GRP75 to promote its apoptosis signaling pathway

**DOI:** 10.1038/s41598-023-41196-0

**Published:** 2023-08-28

**Authors:** Jinghong Xu, Takuya Hashino, Reiji Tanaka, Koichiro Kawaguchi, Hideki Yoshida, Takao Kataoka

**Affiliations:** 1 Department of Applied Biology, Kyoto Institute of Technology, Matsugasaki, Sakyo-ku, Kyoto, 606-8585 Japan; 2 Biomedical Research Center, Kyoto Institute of Technology, Matsugasaki, Sakyo-ku, Kyoto, 606-8585 Japan

**Keywords:** Cell biology, Molecular biology

## Abstract

The BCL-2 family protein BCL-RAMBO, also known as BCL2-like 13, anchors at the outer mitochondrial membrane and regulates apoptosis, mitochondrial fragmentation, and mitophagy. However, the mechanisms underlying the proapoptotic role of BCL-RAMBO remain unclear. In the present study, we demonstrated that BCL-RAMBO interacted with glucose-regulated protein 75 (GRP75), also known as heat shock protein family A member 9, and mortalin using co-immunoprecipitation and glutathione *S*-transferase-based pull-down assays. BCL-RAMBO interacted with GRP75 via its No BCL-2 homology domain. The interaction between BCL-RAMBO and GRP75 was confirmed by genetic interactions in *Drosophila* because a rough eye phenotype caused by the ectopic expression of BCL-RAMBO was partially suppressed by mutations in *Hsc70-5*, a mammalian GRP75 ortholog. In human embryonic kidney 293T cells, the co-expression of BCL-RAMBO and GRP75 facilitated an elevation in executioner caspase activity and poly (ADP-ribose) polymerase 1 (PARP-1) cleavage. In contrast, the knockdown of GRP75 suppressed elevated executioner caspase activity and PARP-1 cleavage in BCL-RAMBO-transfected cells. The mitochondrial release of cytochrome *c* induced by BCL-RAMBO was also attenuated by the knockdown of GRP75. These results indicate that GRP75 interacts with BCL-RAMBO and plays a crucial role in the BCL-RAMBO-dependent apoptosis signaling pathway.

## Introduction

Apoptosis is one of the most important cell death programs and functions to maintain homeostasis by eliminating abnormal or damaged cells in higher organisms^1^. Dysfunctional apoptosis pathways have been linked to many human diseases, including cancer, neurodegenerative disorders, autoimmune diseases, and heart diseases^2^. Apoptosis is mainly divided into two categories: intrinsic and extrinsic pathways^3,4^. Intrinsic apoptosis, also known as mitochondria-dependent apoptosis, is generally regulated by BCL-2 family proteins, which share sequence homology within conserved regions known as the BCL-2 homology (BH) domain^5–7^. The BCL-2 family of proteins regulate mitochondrial outer membrane permeability (MOMP), which mediates the release of multiple proteins (e.g. cytochrome *c*) that are normally confined to the intermembrane space of mitochondria^8–10^. Upon its release into the cytosol, cytochrome *c* binds to apoptotic peptidase activating factor 1 and induces the formation of apoptosomes, which activate the initiator procaspase-9^11,12^. Active caspase-9 then directly cleaves and converts executioner procaspases-3 and -7 into their active forms^13,14^.

BCL-RAMBO, also known as BCL2-like 13, was initially discovered as a pro-apoptotic protein^15^. Many studies have demonstrated that BCL-RAMBO controls cell death, mitochondria fragmentation, and mitophagy^16,17^. BCL-RAMBO consists of an N-terminal BH domain, a No BH (BHNo) domain, a transmembrane (TM) domain, and a short cytoplasmic tail^15^. In contrast to other pro-apoptotic BCL-2 family proteins, the pro-apoptotic activity of BCL-RAMBO depends on the BHNo domain and TM domain instead of the BH domain^15^. Previous studies demonstrated that BCL-RAMBO did not bind to anti-apoptotic and pro-apoptotic BCL-2 family proteins^15,18^; however, it was shown to interact with multiple mammalian proteins, adenine nucleotide translocases (ANT)^19,20^, ceramide synthases (CERS)^21^, microtubule-associated protein 1 light chain 3 (MAP1LC3)^22,23^, phosphoglycerate mutase 5 (PGAM5)^24^, unc-51-like kinase (ULK1)^25^, and voltage-dependent anion channels (VDAC)^20^, as well as the bacterial protein SidF (*Legionella pneumophila*)^26^. ANT, PGAM5, and VDAC have been reported to regulate BCL-RAMBO-mediated apoptosis^19,20,24^, while MAP1LC3, PGAM5, and ULK1 were shown to control BCL-RAMBO-mediated mitophagy^22,24,25^.

Glucose-regulated protein 75 (GRP75), also known as heat shock protein (HSP) family A member 9 or mortalin, belongs to the HSP70 family and interacts with several binding partners^27–29^. GRP75 predominantly resides in mitochondria at which it performs multiple functions, including the import of mitochondrial proteins, while it also localizes to other cellular compartments, including the endoplasmic reticulum (ER), cytoplasmic vesicles, and cytosol^27–29^. VDAC1 was previously reported to interact with GRP75^30^, and was subsequently shown to be linked to the ER Ca^2+^ channel inositol 1,4,5-triphosphate receptor (IP3R) through GRP75^31^. Mitochondria-associated membranes (MAMs) are contact sites between mitochondria and the ER, and exert various biological functions, including lipid metabolism and calcium signaling^32,33^. Accumulating evidence reveals that the IP3R-GRP75-VDAC1 complex localizes at MAMs and mediates the transfer of Ca^2+^ from the ER to mitochondria^32,33^.

We previously reported that BCL-RAMBO and VDAC cooperatively promoted caspase activity in human cultured cells^20^. We also demonstrated that VDAC1 interacted with BCL-RAMBO^20^. The mechanisms by which BCL-RAMBO and VDAC promote caspase activation remain unclear. In the present study, we attempted to test the hypothesis that GRP75 is involved in the BCL-RAMBO-dependent apoptosis signaling pathway. We initially found that BCL-RAMBO interacted with GRP75 using a co-immunoprecipitation assay. We also revealed that BCL-RAMBO interacted and cooperated with GRP75 to promote its apoptosis signaling pathway.

## Results

### GRP75 interacted with BCL-RAMBO via its BHNo domain

**Figure 1:**
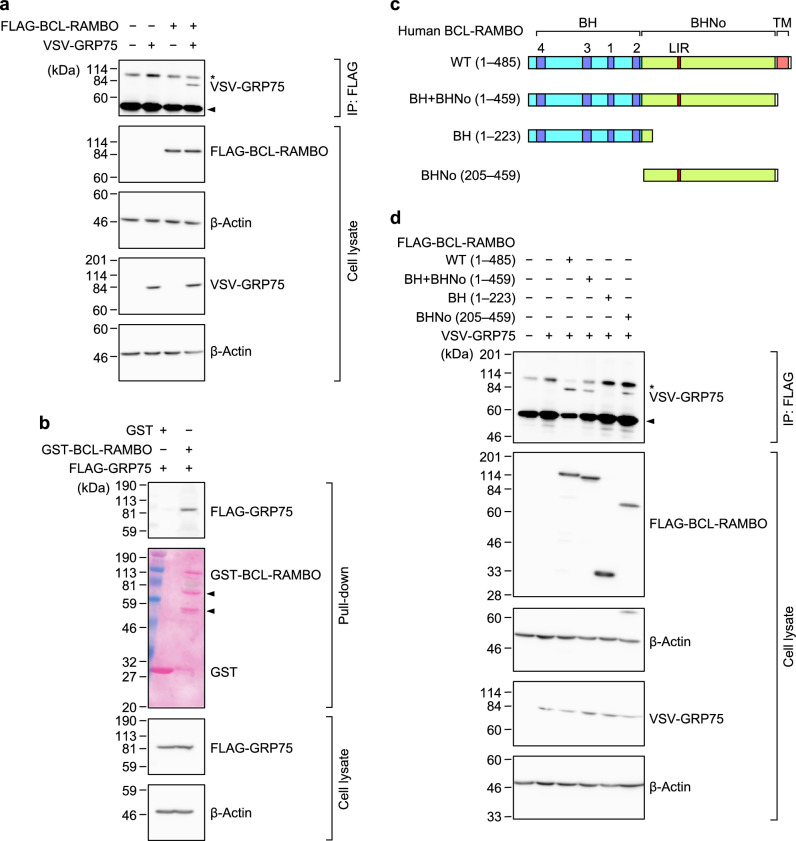
GRP75 interacted with BCL-RAMBO via its BHNo domain. (**a**) 293T cells were transfected without (−) or with (+) expression vectors encoding FLAG-BCL-RAMBO and/or VSV-GRP75 for 18 h. Cell lysates were subjected to immunoprecipitation (IP) and assessed by Western blotting. The asterisk indicates non-specific bands. The arrow corresponds to IgG heavy chains. β-actin (n = 2) or γ1-actin (n = 1) was used as the loading control. Representative blots, in which β-actin was used as the loading control, from three independent experiments are shown. Original blots are presented in Supplementary Fig. S1. (**b**) 293T cells were transfected with (+) expression vectors encoding FLAG-GRP75 for 18 h. Cell lysates were incubated without (−) or with (+) the GST protein or GST-BCL-RAMBO protein together with Glutathione Sepharose beads overnight. Pull-down fractions and cell lysates were assessed by Western blotting. Arrows indicate cleaved forms of the GST-BCL-RAMBO protein. Ponceau S staining was used to detect the GST protein and GST-BCL-RAMBO protein. Original blots and gels are presented in Supplementary Fig. S2. Representative results from three independent experiments are shown. (**c**) Structures of BCL-RAMBO and its deletion mutants. BH: BCL-2 homology motifs, BHNo: No BH motif domain, TM: transmembrane domain, LIR: MAP1LC3-interacting region. (**d**) 293T cells were transfected without (−) or with (+) expression vectors encoding FLAG-BCL-RAMBO mutants and VSV-GRP75 for 18 h. Cell lysates were subjected to immunoprecipitation (IP) and assessed by Western blotting. The asterisk indicates non-specific bands. The arrow corresponds to IgG heavy chains. Original blots are presented in Supplementary Fig. S3. Representative blots from three independent experiments are shown.

To verify the interaction between BCL-RAMBO and GRP75, we overexpressed FLAG-BCL-RAMBO and VSV-GRP75 in 293T cells by transient transfection using pCR3 expression vectors, and performed co-immunoprecipitation assays using anti-FLAG antibody-conjugated agarose beads. VSV-GRP75 was co-immunoprecipitated with FLAG-BCL-RAMBO (Fig. 1a). To confirm this, we also performed pull-down assays using the glutathione *S*-transferase (GST) protein and GST-BCL-RAMBO protein, both of which were produced in *Escherichia coli*, and cell lysates prepared from 293T cells transiently expressing FLAG-GRP75. The GST-BCL-RAMBO protein, but not the GST protein, pulled down FLAG-GRP75 (Fig. 1b). These results showed that GRP75 physically interacted with BCL-RAMBO.

BCL-RAMBO contains four N-terminal BH motifs, a BHNo domain, and a C-terminal TM domain (Fig. 1c)^15^. To identify the interacting regions of BCL-RAMBO and GRP75, we used a series of deletion mutants of BCL-RAMBO (Fig. 1c). FLAG-tagged BCL-RAMBO mutants and VSV-GRP75 were transiently expressed in 293T cells. BCL-RAMBO WT (1–485), BH + BHNo (1–459), and BHNo (205–459), but not BH (1–223), co-immunoprecipitated with GRP75 (Fig. 1d). These results indicated that GRP75 interacted with BCL-RAMBO via its BHNo domain.

### *Hsc70-5*, a mammalian GRP75 ortholog, genetically interacted with BCL-RAMBO

**Figure 2:**
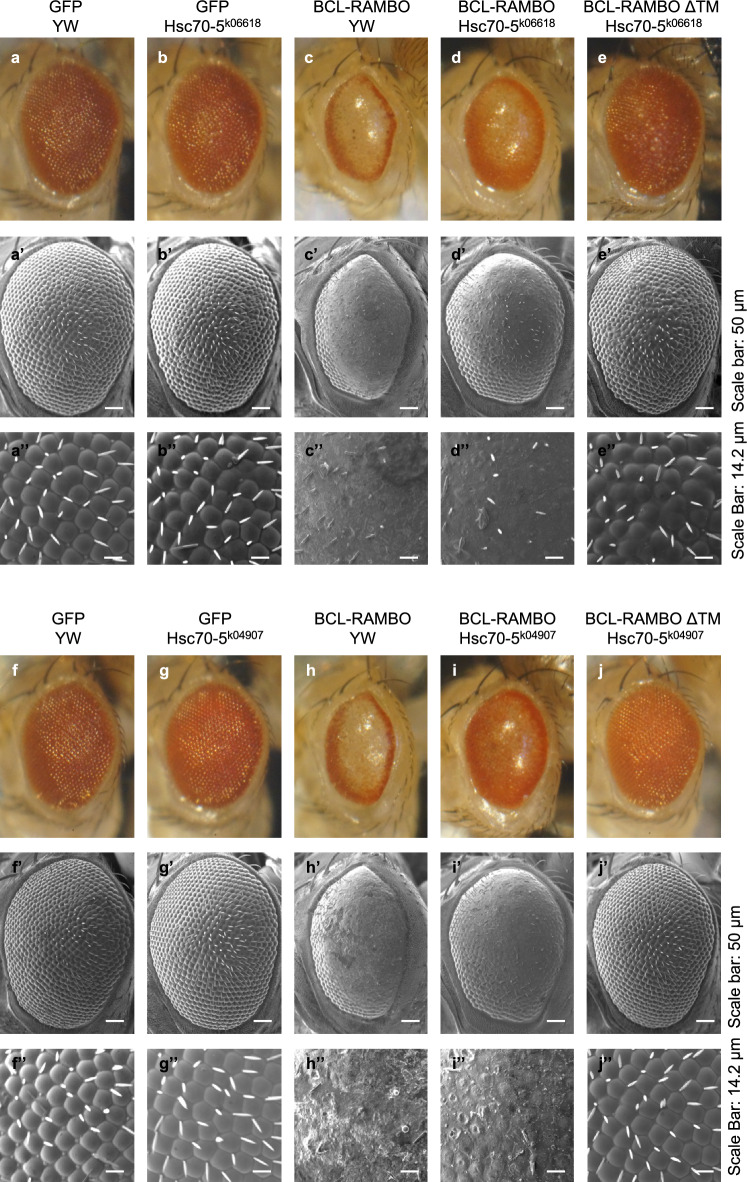
The aberrant rough eye phenotype caused by the ectopic expression of *human BCL-RAMBO* was attenuated by *Hsc70-5* mutations in *Drosophila*. (**a**,**a′**,**a″**) *GMR-GAL4/*+; *UAS-GFP/yw*;+, (**b**,**b′**,**b″**) *GMR-GAL4/*+; *UAS-GFP/Hsc70-5*^*k06618*^;+, (**c**,**c′**,**c″**) *GMR-GAL4/*+; *UAS-BCL-RAMBO/yw*;+, (**d**,**d′**,**d″**) *GMR-GAL4/*+; *UAS-BCL-RAMBO/Hsc70-*5^*k06618*^;+, (**e**,**e′**,**e″**) *GMR-GAL4/*+; *UAS-BCL-RAMBO ΔTM/Hsc70-5*^*k06618*^;+. (**f**,**f′**,**f″**) *GMR-GAL4/*+; *UAS-GFP/yw*;+, (**g**,**g′**,**g″**) *GMR-GAL4/*+; *UAS-GFP/Hsc70-5*^*K04907*^;+, (**h**,**h′**,**h″**) *GMR-GAL4/*+; *UAS-BCL-RAMBO/yw*;+, (**i**,**i′**,**i″**) *GMR-GAL4/*+; *UAS-BCL-RAMBO/Hsc70-5*^*k04907*^;+, (**j**,**j′**,**j″**) *GMR-GAL4/*+; *UAS-BCL-RAMBO ΔTM/Hsc70-5*^*k04907*^;+. The morphology of F1 fly eyes was observed by light microscopy (**a**–**e** and **f**–**j**) and scanning electron microscopy (SEM) (**a′**–**e′**,**a″**–**e″**,**f′**–**j′**, and **f″**–**j″**). Representative results from three independent experiments are shown. Scale bars in (**a′**–**e′**) and (**f′**–**j′**): 50 µm. Scale bars in (**a″**–**e″**) and (**f″**–**j″**): 14.2 µm.

We previously showed that the ectopic expression of *BCL-RAMBO*, but not *BCL-RAMBO ΔTM*, induced apoptosis and caused aberrant rough eye phenotypes in *Drosophila*^18^. To allow the ectopic expression of green fluorescence protein (GFP) and BCL-RAMBO in *Drosophila* eyes, UAS-*GFP* and UAS-*BCL-RAMBO* fly lines were crossed with *glass multiple reporter (GMR)-GAL4* fly lines. The morphology of adult eyes was normal in fly lines expressing GFP (Fig. 2a–a″,f–f″). Consistent with our previous findings^18^, BCL-RAMBO caused a loss of pigmentation, a reduction in eye sizes, and marked morphological changes in ommatidia (Fig. 2c–c″,h–h″). *Hsc70-5* is an ortholog of mammalian *GRP75*. The Hsc70-5 mutations, *Hsc70-5*^*k06618*^ and *Hsc70-5*^*k04907*^, partly rescued the reduction in pigmentation in BCL-RAMBO fly lines (Fig. 2d–d″,i–i″), but did not affect the morphology of adult eyes in fly lines expressing GFP (Fig. 2b–b″,g–g″) and BCL-RAMBO ΔTM (Fig. 2e–e″,j–j″). These results showed that *Hsc70-5* genetically interacted with *BCL-RAMBO* in *Drosophila.*

### GRP75 cooperated with BCL-RAMBO to promote the activation of executioner caspases, while GRP75 knockdown attenuated the activation of executioner caspases induced by BCL-RAMBO

**Figure 3:**
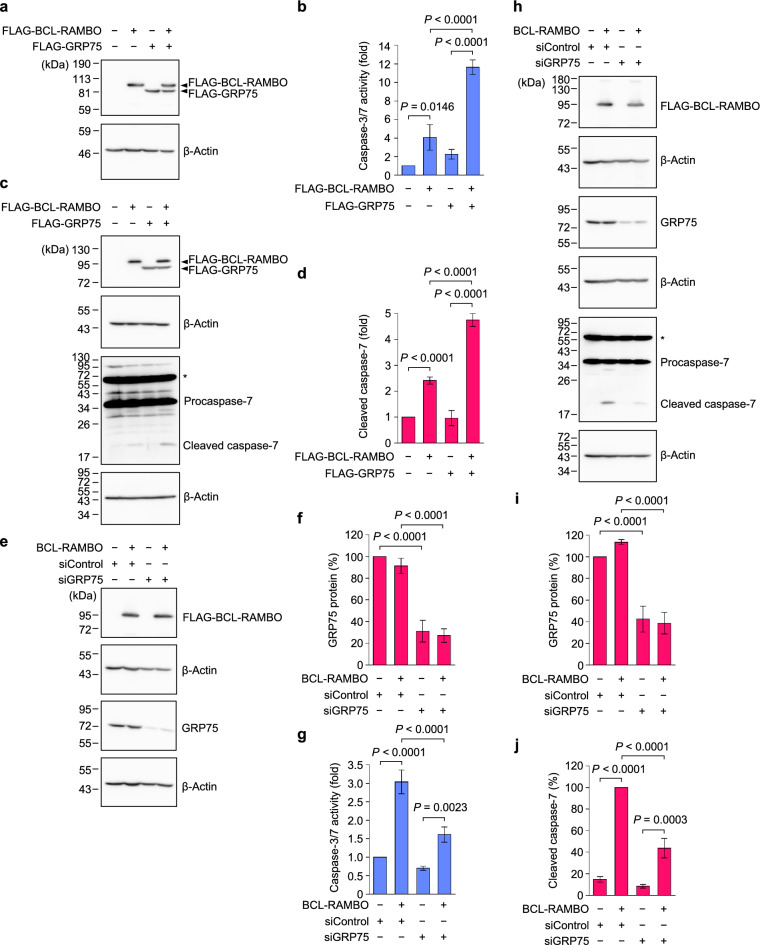
BCL-RAMBO and GRP75 cooperatively promoted the activation of executioner caspases, while GRP75 knockdown suppressed the activation of executioner caspases induced by BCL-RAMBO. (**a**,**b**) 293T cells were transfected without (−) or with (+) expression vectors encoding FLAG-BCL-RAMBO and/or FLAG-GRP75, together with an expression vector encoding the cytomegalovirus promoter-driven *Renilla* luciferase reporter, for 18 h. Cell lysates were assessed by Western blotting (**a**) and caspase-3/7 activity assay (**b**). Original blots are presented in Supplementary Fig. S4. Representative blots from three independent experiments are shown. *Renilla* luciferase activity was used to normalize caspase-3/7 activity. Caspase-3/7 activity (fold) is presented as the mean ± S.E. of three independent experiments. (**c**,**d**) 293T cells were transfected without (−) or with (+) expression vectors encoding FLAG-BCL-RAMBO and/or FLAG-GRP75 for 16 h. Cell lysates were assessed by Western blotting (**c**). The asterisk indicated additional bands detected by the anti-caspase-7 antibody. Original blots are presented in Supplementary Fig. S5. Representative blots from three independent experiments are shown. The amount of β-actin was used to normalize cleaved caspase-7. Cleaved caspase-7 (fold) is presented as the mean ± S.E. of three independent experiments (**d**). (**e**–**g**) 293T cells were transfected without (−) or with (+) siControl or siGRP75 for 24 h, and then transfected without (−) or with (+) expression vectors encoding FLAG-BCL-RAMBO together with an expression vector encoding the cytomegalovirus promoter-driven *Renilla* luciferase reporter for 16 h. Cell lysates were assessed by Western blotting (**e**) and caspase-3/7 activity assay (**g**). Original blots are presented in Supplementary Fig. S6. Representative blots from three independent experiments are shown. The amount of β-actin was used to normalize that of GRP75. GRP75 protein (%) is presented as the means ± S.E. of three independent experiments (**f**). *Renilla* luciferase activity was used to normalize caspase-3/7 activity. Caspase-3/7 activity (fold) is presented as the means ± S.E. of three independent experiments (**g**). (**h**–**j**) 293T cells were transfected without (−) or with (+) siControl or siGRP75 for 24 h, and then transfected without (−) or with (+) expression vectors encoding FLAG-BCL-RAMBO for 16 h. Cell lysates were assessed by Western blotting (**h**). The asterisk indicates additional bands detected by the anti-caspase-7 antibody. Original blots are presented in Supplementary Fig. S7. Representative blots from three independent experiments are shown. The amount of β-actin was used to normalize those of GRP75 and cleaved caspase-7. GRP75 protein (%) (**i**) and cleaved caspase-7 (%) (**j**) are presented as the means ± S.E. of three independent experiments. GRP75 protein in siControl-transfected 293T cells is set to 100%. Cleaved caspase-7 in siControl- and BCL-RAMBO-transfected 293T cells is set to 100%. *P* values less than 0.05 are shown.

BCL-RAMBO has been shown to induce apoptosis when transiently expressed in cultured cell lines^15,18,19,26,34^. To examine the effects of GRP75 on BCL-RAMBO-induced caspase activation, 293T cells were transiently transfected with expression vectors encoding FLAG-BCL-RAMBO and FLAG-GRP75 for 18 h. FLAG-BCL-RAMBO and FLAG-GRP75 were expressed at similar levels in 293T cells (Fig. 3a). Under these conditions, BCL-RAMBO augmented caspase-3/7 activity, while GRP75 had no effects (Fig. 3b). In contrast, GRP75 promoted the augmentation of caspase-3/7 activity induced by BCL-RAMBO (Fig. 3b).

We further investigated the activation of procaspase-7 using Western blotting. FLAG-BCL-RAMBO and FLAG-GRP75 were detected to a similar degree when expressed in 293T cells for 16 h (Fig. 3c). Consistent with caspase-3/7 activity (Fig. 3b), BCL-RAMBO, but not GRP75, augmented the cleavage of procaspase-7 into the active p20 subunit (Fig. 3c,d). GRP75 promoted the augmentation of procaspase-7 cleavage induced by BCL-RAMBO (Fig. 3c,d). These results showed that GRP75 cooperated with BCL-RAMBO to promote the activation of executioner caspases.

We investigated whether the knockdown of GRP75 attenuated the BCL-RAMBO-induced apoptosis signaling pathway. Treatment of 293T cells with siGRP75 resulted in an approximately 70% reduction in the GRP75 protein compared to siControl (Fig. 3e,f). To induce sufficient activation of executioner caspases by BCL-RAMBO itself, we used a larger amount of DNA in the transient transfection experiments. Treatment with siGRP75 reduced the BCL-RAMBO-induced increase in caspase-3/7 activity (Fig. 3g). Caspase-7 cleavage was then assessed by Western blotting. The amount of cleaved caspase-7 was augmented by BCL-RAMBO in siControl-treated 293T cells (Fig. 3h,j). The treatment of 293T cells with siGRP75 resulted in a decrease in the GRP75 protein from that with siControl by approximately 60% (Fig. 3h,i). Under these conditions, the augmentation of cleaved caspase-7 caused by BCL-RAMBO was attenuated by the siGRP75 treatment (Fig. 3h,j). These results indicated that GRP75 knockdown attenuated BCL-RAMBO-induced executioner caspase activation.

### GRP75 cooperated with BCL-RAMBO to promote PARP-1 cleavage, while GRP75 knockdown attenuated BCL-RAMBO-induced PARP-1 cleavage

**Figure 4:**
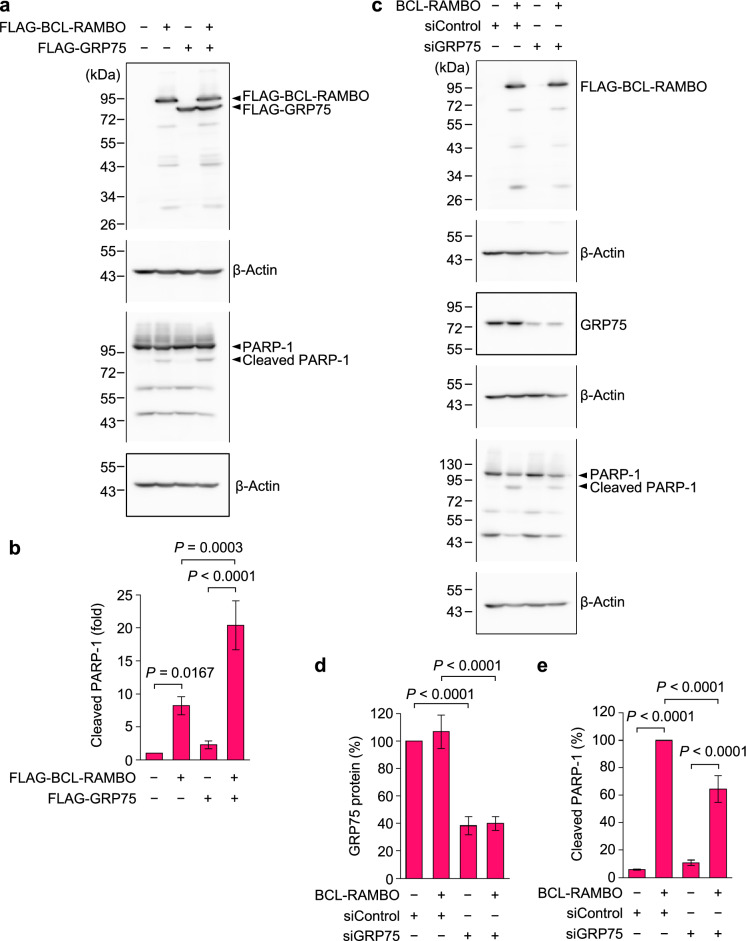
BCL-RAMBO and GRP75 cooperatively promoted PARP-1 cleavage, whereas GRP75 knockdown suppressed the BCL-RAMBO-induced PARP-1 cleavage. (**a**,**b**) 293T cells were transfected without (−) or with (+) expression vectors encoding FLAG-BCL-RAMBO and/or FLAG-GRP75 for 24 h. Whole cell lysates were assessed by Western blotting (**a**). Original blots are presented in Supplementary Fig. S8. Representative blots from three independent experiments are shown. The amount of β-actin was used to normalize that of cleaved PARP-1. Cleaved PARP-1 (fold) is presented as the mean ± S.E. of three independent experiments (**b**). (**c–e**) 293T cells were transfected without (−) or with (+) siControl or siGRP75 for 24 h, and then transfected without (−) or with (+) expression vectors encoding FLAG-BCL-RAMBO for 24 h. Whole cell lysates were assessed by Western blotting (**c**). Original blots are presented in Supplementary Fig. S9. Representative blots from three independent experiments are shown. The amount of β-actin was used to normalize those of GRP75 (**d**) and cleaved PARP-1 (**e**). GRP75 protein (%) (**d**) and cleaved PARP-1 (%) (**e**) are presented as the means ± S.E. of three independent experiments. GRP75 protein in siControl-transfected 293T cells is set to 100%. Cleaved PARP-1 in siControl- and BCL-RAMBO-transfected 293T cells is set to 100%. *P* values less than 0.05 are shown.

Poly (ADP-ribose) polymerase 1 (PARP-1) (113 kDa) is known to be one of the cellular caspase substrates, and is cleaved into 89 kDa fragments by caspase-3 and caspase-7 during apoptosis^35,36^. We further investigated the cleavage of PARP-1 by Western blotting. Transfection of 293T cells with BCL-RAMBO for 24 h increased PARP-1 cleavage, whereas BCL-RAMBO-induced PARP-1 cleavage was promoted by co-transfection with GRP75 (Fig. 4a,b). These results demonstrated that GRP75 cooperated with BCL-RAMBO to promote the cleavage of the caspase substrate PARP-1.

We investigated the effect of GRP75 knockdown on BCL-RAMBO-induced PARP-1 cleavage. Treatment of 293T cells with siGRP75 reduced the amount of GRP75 protein by approximately 60% (Fig. 4c,d). BCL-RAMBO-induced PARP-1 cleavage was reduced in siGRP75-treated cells (Fig. 4c,e). These results indicated that GRP75 knockdown attenuated the BCL-RAMBO-induced PARP-1 cleavage.

### The knockdown of GRP75 attenuated the release of cytochrome *c* into the cytosol

**Figure 5:**
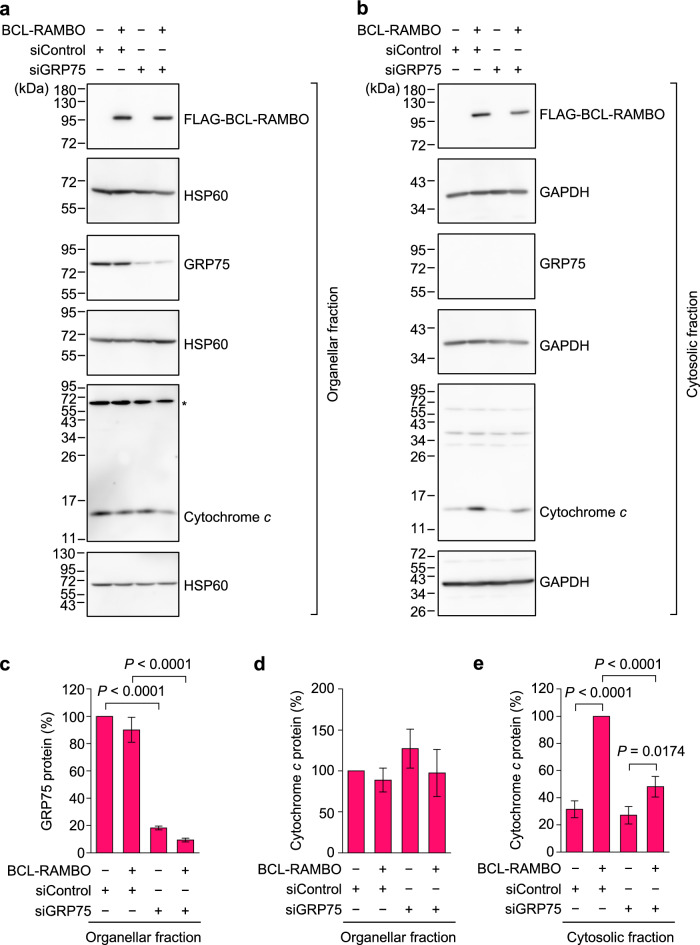
The knockdown of GRP75 attenuated the mitochondrial release of cytochrome *c* induced by BCL-RAMBO. (**a**–**e**) 293T cells were transfected with siControl or siGRP75 for 24 h and then transfected without (−) or with (+) expression vectors encoding FLAG-BCL-RAMBO for 16 h. Cell lysates were separated into the organellar fraction (**a**) and cytosolic fraction (**b**), followed by Western blotting. The asterisk indicated non-specific bands detected by the anti-cytochrome *c* antibody. Original blots in (**a**) and (**b**) are presented in Supplemental Figs. S10 and S11, respectively. Representative blots from three independent experiments are shown. The amount of HSP60 was used to normalize that of GRP75 in the organellar fraction. The amounts of cytochrome *c* protein in the organellar and cytosolic fractions were normalized to those of HSP60 and GAPDH. GRP75 protein in the organellar fraction (%) (**c**), cytochrome *c* protein in the organellar fraction (%) (**d**), and cytochrome *c* protein in the cytosolic fraction (%) (**e**) are presented as the means ± S.E. of three independent experiments. GRP75 protein and cytochrome *c* protein in the organellar fraction of siControl-transfected 293T cells were set to 100%. Cytochrome *c* protein in the cytosolic fraction of siControl- and BCL-RAMBO-transfected 293T cells was set to 100%.* P* values less than 0.05 are shown.

In mitochondrial apoptosis, the release of cytochrome *c* into the cytosol is essential for the activation of caspases^11,12^. We previously showed that BCL-RAMBO induced the mitochondrial release of cytochrome *c* in the cytosol^15,18^. To evaluate the subcellular localization of cytochrome *c*, cell lysates were separated into cytosolic, nuclear, and organellar fractions by successive treatments with digitonin and Triton X-100, followed by centrifugation. The GRP75 protein was recovered in the organellar fraction, but not in the cytosolic fraction (Fig. 5a,b). The treatment of 293T cells with siGRP75 diminished the amount of the GRP75 protein from that with siControl by more than 80% (Fig. 5a,c). Cytochrome *c* levels in the organellar fraction were not markedly affected by BCL-RAMBO or siGRP75 (Fig. 5a,d). BCL-RAMBO augmented the amount of cytochrome *c* in the cytosolic fraction of siControl-treated cells (Fig. 5b,e). The BCL-RAMBO-induced augmentation of cytosolic cytochrome *c* was markedly diminished in siGRP75-treated cells (Fig. 5b,e). These results showed that the knockdown of GRP75 attenuated the BCL-RAMBO-induced release of cytochrome *c* into the cytosol.

### BCL-RAMBO formed a complex with GRP75 and VDAC1

**Figure 6:**
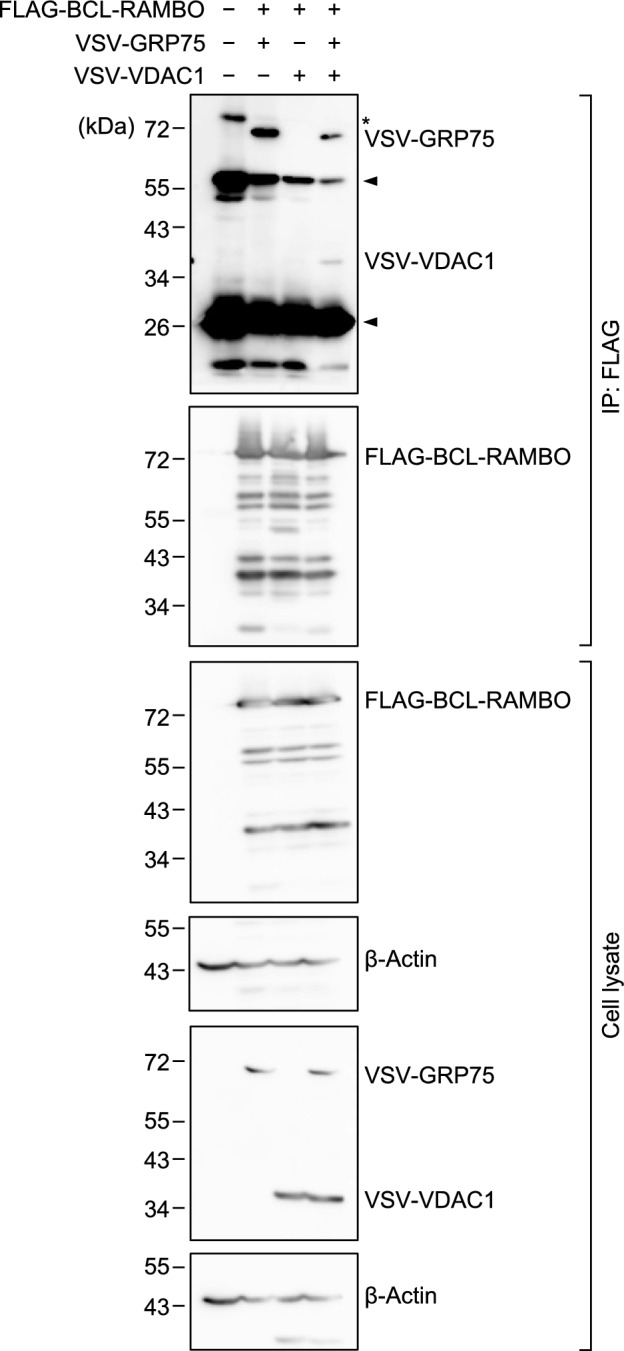
BCL-RAMBO formed a complex with GRP75 and VDAC1. 293T cells were transfected without (−) or with (+) expression vectors encoding FLAG-BCL-RAMBO, VSV-GRP75, and/or VSV-VDAC1 for 18 h. Cell lysates were subjected to immunoprecipitation (IP) and assessed by Western blotting. The asterisk indicates non-specific bands. The arrows correspond to IgG chains. Original blots are presented in Supplementary Fig. S12. Representative blots from three independent experiments are shown.

We showed that GRP75 interacted with BCL-RAMBO via its BHNo domain (Fig. 1d). VDAC1 has been previously reported to interact with GRP75^30^. To test whether the complex formed with BCL-RAMBO and GRP75 contains additional factors such as VDAC1, we transfected 293T cells with expression vectors encoding FLAG-BCL-RAMBO, together with VSV-GRP75 and VSV-VDAC1, and cell lysates were used for immunoprecipitation assay using anti-FLAG antibody beads. As shown in Fig. 1a, VSV-GRP75 was immunoprecipitated with FLAG-BCL-RAMBO (Fig. 6). In contrast, VSV-VDAC1 was barely immunoprecipitated with FLAG-BCL-RAMBO (Fig. 6). However, VSV-VDAC1 was immunoprecipitated with FLAG-BCL-RAMBO when VSV-GRP75 was co-transfected (Fig. 6). These results suggest that GRP75 mediates the interaction between BCL-RAMBO and VDAC1.

## Discussion

The overexpression of BCL-RAMBO has been shown to induce apoptosis in human embryonic kidney 293T cells, human breast cancer MCF-7 cells, and human prostate cancer PC3 cells^15,19,26^. A previous study demonstrated that BCL-RAMBO promoted taxol-induced cell death in 293A cells and etoposide-induced cell death in human cervical cancer HeLa cells^34^. In *Drosophila* S2 cells, the ectopic expression of BCL-RAMBO induced apoptosis^18^. Collectively, these findings showed that BCL-RAMBO is a pro-apoptotic protein capable of inducing apoptosis in multiple cell lines. In the present study, we identified GRP75 as an emerging partner capable of interacting with BCL-RAMBO. GRP75 promoted BCL-RAMBO-induced caspase activity and PARP-1 cleavage, while its knockdown attenuated BCL-RAMBO-induced caspase activity, PARP-1 cleavage, and the mitochondrial release of cytochrome *c*. The present results revealed that GRP75 functions as an important regulator of the BCL-RAMBO-dependent apoptosis signaling pathway.

The present study revealed that GRP75 interacted with BCL-RAMBO. In addition to the import of mitochondrial proteins, GRP75 exerts various functions by interacting with several binding partners^27–29^. Closely related to the present study, GRP75 was shown to become a bridge between the ER channel protein IP3R and the mitochondrial channel protein VDAC1^31^. The IP3R-GRP75-VDAC1 complex is present in MAMs and transfers Ca^2+^ from the ER and mitochondria^37^. We previously showed that the GST-BCL-RAMBO protein interacted with FLAG-VDAC1 expressed in 293T cells^20^. In this study, the immunoprecipitation assay showed that BCL-RAMBO formed a complex with GRP75 and VDAC1. Taken together, the present results suggest that BCL-RAMBO associates with the IP3R-GRP75-VDAC1 complex and is an additional component that regulates its biological activity.

BCL-RAMBO has been shown to mediate pro-apoptotic activity via the BHNo domain anchored at the mitochondrial outer membrane, whereas the BH domain of BCL-RAMBO is not necessarily essential for pro-apoptotic activity^15^. To date, BCL-RAMBO has been shown to induce MOMP in a manner that is independent of its direct interactions with other BCL-2 family proteins. In the present study, we showed that GRP75 interacted with BCL-RAMBO via its BHNo domain. BCL-RAMBO and GRP75 cooperatively promoted caspase activation and PARP-1 cleavage, while the knockdown of GRP75 attenuated the BCL-RAMBO-induced mitochondrial release of cytochrome *c*, caspase activation, and PARP-1 cleavage. Therefore, GRP75 appears to interact with BCL-RAMBO, thereby promoting MOMP. However, future studies are needed to elucidate the mechanism by which BCL-RAMBO and GRP 75 induce cytochrome *c* release from mitochondria, including the multimerization of BAX and BAK.

GRP75 has been identified as an important regulator of apoptosis, which is mediated by the IP3R-GRP75-VDAC1 complex^38,39^. Adriamycin and angiotensin II were found to increase the expression of IP3R, GRP75, VDAC1, and mitochondrial calcium uniporter, and thereby induced apoptosis in mouse podocytes, while the knockdown of GRP75 inhibited adriamycin- and angiotensin II-induced apoptosis^38^. The knockdown of GRP75 also attenuated elevated mitochondrial Ca^2+^ levels in retinal microvascular endothelial cells (RMECs) treated with high glucose or advanced glycosylation end products, which represent a cellular model for diabetic retinopathy^39^. BAPTA-AM, an intracellular calcium chelator, prevented apoptosis induced by tunicamycin, an ER stress inducer, in RMECs^39^. These findings indicate that GRP75 regulates mitochondrial Ca^2+^ levels and thereby promotes Ca^2+^-dependent apoptosis. Ca^2+^ plays an important role in the regulation of mitochondria metabolism; low Ca^2+^ levels are essential for maintaining the optimal rates of ATP production, while excess Ca^2+^ levels induce the loss of mitochondrial functions^40,41^. The mitochondrial influx of Ca^2+^ triggers apoptosis, necrosis, and autophagy^40,41^. Further studies are needed to confirm whether BCL-RAMBO regulates mitochondrial Ca^2+^-dependent processes.

In conclusion, the present results provide novel insights into the mechanisms underlying apoptotic signaling by BCL-RAMBO. To date, the physiological stimuli that induce apoptosis via BCL-RAMBO have not been well understood. Further studies are needed to link BCL-RAMBO to the IP3R-GRP75-VDAC1 complex and elucidate the role of GRP75 in BCL-RAMBO-induced apoptosis under physiological and pathological settings.

## Materials and methods

### Cell lines

Human embryonic kidney 293T cells (RCB2202) were provided by Cell Bank, the RIKEN BioResource Research Center (Tsukuba, Japan). 293T cells, which were used for experiments to induce apoptosis, were described in our previous studies^15,42^. 293T cells were maintained with Dulbecco’s Modified Eagle’s medium (Thermo Fisher Scientific, Waltham, MA, USA) supplemented with heat-inactivated fetal calf serum (Sigma-Aldrich, St. Louis, MO, USA) and penicillin–streptomycin mixed solution (Nacalai Tesque, Kyoto, Japan) at 37 °C in 5% CO_2_.

### Fly stocks

Fly stocks were kept at 25 °C and fed food containing 0.7% agar, 10% glucose, 3% rice bran, and 4% dry yeast. The UAS-*BCL-RAMBO* and UAS-*BCL-RAMBO ΔTM* fly lines were generated in our previous study^18^. Fly lines carrying *GMR-GAL4* were previously described^43^. *Hsc70-5*^*k06618*^*/CyO* (102491) fly lines were obtained from the Kyoto Stock Center, Kyoto Institute of Technology (Kyoto, Japan). The *Hsc70-5*^*k04907*^*/CyO* (10557) and UAS-*GFP* (9331) fly lines were obtained from the Bloomington Drosophila Stock Center, Indiana University (Bloomington, IN, USA).

### Plasmids

pCR3 expression vectors encoding FLAG-tagged wild-type human BCL-RAMBO (1–485) (GeneBank accession: AF325209), BCL-RAMBO (1–459), BCL-RAMBO (1–223), BCL-RAMBO (205–459), and pGEX-6-P1 expression vectors encoding human BCL-RAMBO were previously described^15,24^. A plasmid encoding human GRP75 cDNA (clone: HEP01066; GenBank accession: AK222758) was a kind gift from Sumio Sugano and Akiko Tanaka. Full-length GRP75 (1–679) was subcloned into pCR3 expression vectors encoding an N-terminal FLAG-tag and VSV-tag.

### Antibodies

Mouse monoclonal antibodies to FLAG (IE6; FUJIFILM Wako Pure Chemical Corporation, Osaka, Japan), VSV-G (P5D4; Santa Cruz Biotechnology, Dallas, TX, USA), the VSV glycoprotein (P5D4, SAB4200695; Sigma-Aldrich), GRP75 (D-9; Santa Cruz Biotechnology), cytochrome *c* (7H8.2C12; BD Biosciences, Franklin Lakes, NJ, USA), PARP-1 (C2-10; R &D Systems, Minneapolis, MN, USA), γ1-actin (2F3; FUJIFILM Wako Pure Chemical Corporation), β-actin (AC-15; Sigma-Aldrich), and GAPDH (6C5; Santa Cruz Biotechnology), a rat monoclonal antibody to caspase-7 (11E4; Sigma-Aldrich), and a rabbit polyclonal antibody to HSP60 (insect, ADI-SPA-805; Enzo Life Sciences, Farmingdale, NY, USA) were used as primary antibodies. Peroxidase-conjugated goat anti-mouse IgG (H + L) (115-035-146; Jackson ImmunoResearch Laboratories, West Grove, PA, USA), goat anti-rabbit IgG (H + L) (111-035-144; Jackson ImmunoResearch Laboratories), and goat anti-rat IgG (H + L) (J112-035-143; Jackson ImmunoResearch Laboratories) were used as secondary antibodies.

### Microscopic observations

*GMR-GAL4* driver fly lines were crossed with the UAS fly lines. Fly lines were then crossed with *Hsc70-5* mutant fly lines. The compound eyes of adult flies were observed using the stereomicroscope SZX10 (Olympus, Tokyo, Japan) and scanning electron microscope VE-7800 (Keyence, Osaka, Japan).

### Knockdown by siRNA

Predesigned siRNA for GRP75 (SASI_Hs01_00216923; Sigma-Aldrich) and MISSION® siRNA Universal Negative Control #1 (SIC001; Sigma-Aldrich) were used for knockdown experiments. 293T cells were transfected with siControl or siGRP75 (each 30 nM) by Lipofectamine™ RNAiMAX (Thermo Fisher Scientific) and then incubated for 24 h. 293T cells were transfected with the pCR3 expression vector encoding FLAG-BCL-RAMBO by HilyMax transfection reagent (Dojindo, Kumamoto, Japan) and further incubated for 16 h or 24 h.

### Immunoprecipitation

293T cells were transfected with pCR3 expression vectors encoding FLAG-tagged and VSV-tagged target genes using the calcium phosphate method and then incubated for 18 h. 293T cells were washed with ice-cold phosphate-buffered saline (PBS) and lysed with Nonidet P-40 lysis buffer (0.2% NP-40, 150 mM NaCl, 20 mM Tris–HCl (pH 7.4), 10% glycerol, and 2 mM sodium vanadate) containing protease inhibitor cocktail cOmplete™ (Sigma-Aldrich). After centrifugation (15,300×*g*, 5 min), postnuclear cell lysates were collected as supernatants and incubated with Sepharose 4B beads (Sigma-Aldrich) at 4 °C for 1 h. The precleared supernatants were then incubated with anti-FLAG M2 affinity agarose beads (Sigma-Aldrich) at 4 °C for 3 h. Anti-FLAG M2 agarose beads were collected, and then washed at least five times with NP-40 lysis buffer. Immunoprecipitates and precleared cell lysates were separated by SDS-PAGE and analyzed by Western blotting.

### Pull-down assay

The GST protein and GST-BCL-RAMBO protein were prepared by *E. coli* BL21 (DE3) as previously described^20^. 293T cells were transfected with the pCR3 expression vector encoding FLAG-GRP75 using the calcium phosphate method and incubated for 18 h. 293T cells were washed with ice-cold PBS and then lysed with Nonidet P-40 lysis buffer, followed by centrifugation (15,300×*g*, 5 min) to collect supernatants as cell lysates. The GST protein or GST-BCL-RAMBO protein (25 µg each) was incubated with cell lysates (500 µg of proteins) and Glutathione Sepharose® 4B beads at 4 °C overnight. Glutathione Sepharose® 4B beads were washed at least five times with NP-40 wash buffer. Pull-down fractions and cell lysates were separated by SDS-PAGE and analyzed by Western blotting. Ponceau S staining was used to detect the GST protein and GST-BCL-RAMBO protein.

### Western blotting

Cell lysates were prepared as described elsewhere in this report. Whole cell lysates were prepared as previously described^44^. The amount of proteins in cell lysates was measured by CBB solution for Protein Assays (Nacalai Tesque). An equal amount of cell lysates was separated by SDS-PAGE. Gels were transferred to 0.22-µm ClearTrans® nitrocellulose membranes (FUJIFILM Wako Pure Chemical Corporation). After blocking with 5% skim milk in 0.5% Tween 20–PBS (PBS-T) overnight, membranes were treated with primary antibodies in 5% skim milk–PBS-T for 1 h and washed with PBS-T. The membranes were further incubated with secondary antibodies in 5% skim milk–PBS-T for 1 h and then washed with PBS-T. Protein bands were visualized using Amersham™ ECL Western Blotting Detection Reagents (Cytiva, Tokyo, Japan) or ImmunoStar® Zeta (FUJIFILM Wako Pure Chemical Corporation), acquired by Amersham™ Imager 680 (GE Healthcare Japan, Tokyo, Japan) and analyzed by ImageQuant™ TL software version 7.0 (GE Healthcare Japan).

### Caspase assay

293T cells were transiently transfected with pCR3 expression vectors encoding FLAG-tagged target genes together with the pCR3 expression vector encoding cytomegalovirus promoter-driven *Renilla* luciferase to measure transfection efficiencies. After an incubation for 16 h or 18 h, 293T cells were washed with ice-cold PBS and lyzed with digitonin lysis buffer (10 mM Hepes–KOH (pH7.2), 0.1 mM digitonin, 250 mM sucrose, 50 mM NaCl, 2 mM MgCl_2_, 5 mM EGTA, and 1 mM dithiothreitol). Cell lysates were collected as supernatants after centrifugation (15,300×*g*, 5 min). Cell lysates were used to evaluate aminoluciferin-dependent luciferase activity by the Caspase-Glo® 3/7 Assay System (Promega, Madison, WI, USA) and coelenterazine-dependent *Renilla* luciferase activity by a previously reported method^45^. Relative light units were measured by Lumitester C-110 (Kikkoman Biochemifa, Tokyo, Japan). *Renilla* luciferase activity was used to normalize caspase-3/7 activity.

### Subcellular fractionation

293T cells were transiently transfected with siRNAs and pCR3 expression vectors. Transfected cells were washed with ice-cold PBS and then lysed with digitonin lysis buffer containing the protease inhibitor cocktail cOmplete™ on ice for 15 min. Cell lysates were centrifuged (15,300×*g*, 5 min) and then separated into supernatants (cytosolic fraction) and precipitates. Precipitates were washed at least three times with digitonin lysis buffer and then lysed with Triton X-100 lysis buffer containing cOmplete™ on ice for 15 min. Cell lysates were centrifuged (15,300×*g*, 5 min) and separated into supernatants (organellar fraction) and precipitates (nuclear fraction). Cytosolic and organellar fractions were separated by SDS-PAGE and subsequently analyzed by Western blotting. These methods were used to evaluate cytochrome *c* release in human leukemia Jurkat cells and *Drosophila* S2 cells^18,46^.

### Data analysis

Data are presented as the means ± standard error (S.E.). Data analyses were performed by a one-way ANOVA and Tukey’s post-hoc test for multiple comparisons using the KaleidaGraph software version 4.5.1 (Hulinks, Tokyo, Japan).

### Supplementary Information

Supplementary Figures.

## Data Availability

All raw data used in the present study are available upon request from the corresponding author.

## References

[1] Fuchs Y, Steller H (2011). Programmed cell death in animal development and diseases. Cell.

[2] Favaloro B, Allocati N, Graziano V, Di Ilio C, De Laurenzi V (2012). Role of apoptosis in disease. Aging.

[3] D’Arcy MS (2019). Cell death: A review of the major forms of apoptosis, necrosis and autophagy. Cell Biol. Int..

[4] Tang D, Kang R, Berghe TV, Vandenabeele P, Kroemer G (2019). The molecular machinery of regulated cell death. Cell Res..

[5] Adams JM, Cory S (2018). The BCL-2 arbiters of apoptosis and their growing role as cancer targets. Cell Death Differ..

[6] Singh R, Letai A, Sarosiek K (2019). Regulation of apoptosis in health and disease: The balancing act of BCL-2 family proteins. Nat. Rev. Mol. Cell Biol..

[7] Warren CF, Wong-Brown MW, Bowden N (2019). BCL-2 family isoforms in apoptosis and cancer. Cell Death Dis..

[8] Kale J, Osterlund EJ, Andrews DW (2018). BCL-2 family proteins: Changing partners in the dance towards death. Cell Death Differ..

[9] Kalkavan H, Green DR (2018). MOMP, cell suicide as a BCL-2 family business. Cell Death Differ..

[10] Bock FJ, Taito SWG (2020). Mitochondrial as multifaceted regulators of cell death. Nat. Rev. Mol. Cell Biol..

[11] Bratton SB, Salvesen GS (2010). Regulation of the Apaf-1–caspase-9 apoptosome. J. Cell Sci..

[12] Yuan S, Akey CW (2013). Apoptosome structure, assembly, and procaspase activation. Structure.

[13] Mcllwain DR, Berger T, Mak TW (2013). Caspase functions in cell death and disease. Cold Spring Harb. Perspect. Biol..

[14] Man SM, Kanneganti TD (2016). Converging roles of caspases in inflammasome activation, cell death and innate immunity. Nat. Rev. Immunol..

[15] Kataoka T (2001). Bcl-rambo, a novel Bcl-2 homologue that induces apoptosis via its unique C-terminal extension. J. Biol. Chem..

[16] Meng F (2021). BCL2L13: Physiological and pathological meanings. Cell. Mol. Life Sci..

[17] Kataoka T (2022). Biological properties of the BCL-2 family protein BCL-RAMBO, which regulates apoptosis, mitochondrial fragmentation, and mitophagy. Front. Cell Dev. Biol..

[18] Nakazawa M (2016). The human Bcl-2 family member Bcl-rambo localizes to mitochondria and induces apoptosis and morphological aberrations in *Drosophil*a. PLoS ONE.

[19] Kim JY, So KJ, Lee S, Park JH (2012). Bcl-rambo induces apoptosis via interaction with the adenine nucleotide translocator. FEBS Lett..

[20] Matsubara H (2019). The human Bcl-2 family member Bcl-rambo and voltage-dependent anion channels manifest a genetic interaction in *Drosophila* and cooperatively promote the activation of effector caspases in human cultured cells. Exp. Cell Res..

[21] Jensen SA (2014). Bcl2L13 is a ceramide synthase inhibitor in glioblastoma. Proc. Natl. Acad. Sci. U.S.A..

[22] Murakawa T (2015). Bcl-2-like protein 13 is a mammalian Atg32 homologue that mediates mitophagy and mitochondrial fragmentation. Nat. Commun..

[23] Li M, Jia J, Zhang X, Dai H (2020). Selective binding of mitophagy receptor protein Bcl-rambo to LC3/GABARAP family proteins. Biochem. Biophys. Res. Commun..

[24] Hashino T (2022). PGAM5 interacts with Bcl-rambo and regulates apoptosis and mitophagy. Exp. Cell Res..

[25] Murakawa T (2019). A mammalian mitophagy receptor, Bcl2-L-13, recruits the ULK1 complex to induce mitophagy. Cell Rep..

[26] Banga S (2007). *Legionella pneumophila* inhibits macrophage apoptosis by targeting pro-death members of the Bcl2 protein family. Proc. Natl. Acad. Sci. U.S.A..

[27] Londono C, Osorio C, Gama V, Alzate O (2012). Mortalin, apoptosis and neurodegeneration. Biomolecules.

[28] Flachbartová Z, Kovacech B (2013). Mortalin—A multipotent chaperon regulating cellular processes ranging from viral infection to neurodegeneration. Acta Virol..

[29] Esfahanian N, Knoblich CD, Bowman GA, Rezvani K (2023). Mortalin: Protein partner, biological impact, pathological roles, and therapeutic opportunities. Front. Cell Dev. Biol..

[30] Schwarzer C, Barnikol-Watanabe S, Thinnes FP, Hilschmann N (2002). Voltage-dependent anion-selective channel (VDAC) interacts with the dynein light chain Tctex1 and the heat-shock protein PBP74. Int. J. Biochem. Cell Biol..

[31] Szabadkai G (2006). Chaperone-mediated coupling of endoplasmic reticulum and mitochondrial Ca^2+^ channels. J. Cell Biol..

[32] Patergnani S (2011). Calcium signaling around mitochondrial associated membranes (MAMs). Cell Commun. Signal.

[33] Raturi A, Simmen T (2013). Where the endoplasmic reticulum and the mitochondrion tie the knot: The mitochondria-associated membrane (MAM). Biochim. Biophys. Acta.

[34] Yi P (2002). Bcl-rambo beta, a special splicing variant with an insertion of an Alu-like cassette, promotes etoposide- and Taxol-induced cell death. FEBS Lett..

[35] Soldani C, Scovassi AI (2002). Poly(ADP-ribose) polymerase-1 cleavage during apoptosis: An update. Apoptosis.

[36] Chaitanya GV, Alexander JS, Babu PP (2010). PARP-1 cleavage fragments: Signatures of cell-death proteases in neurodegeneration. Cell Commun. Signal..

[37] Xu HX, Cui SM, Zhang YM, Ren J (2020). Mitochondrial Ca^2+^ regulation in the etiology of heart failure: Physiological and pathophysiological implication. Acta Pharmacol. Sin..

[38] Xu H (2018). IP_3_R-Grp75-VDAC1-MCU calcium regulation axis antagonists protect podocytes from apoptosis and decrease proteinuria in an Adriamycin nephropathy rat model. BMC Nephrol..

[39] Li Y (2022). GRP75 modulates endoplasmic reticulum–mitochondria coupling and accelerates Ca^2+^-dependent endothelial cell apoptosis in diabetic retinopathy. Biomolecules.

[40] Giorgi C, Marchi S, Pinton P (2018). The machineries, regulation and cellular functions of mitochondrial calcium. Nat. Rev. Mol. Cell Biol..

[41] Rossi A, Pizzo P, Filadi R (2019). Calcium, mitochondria and cell metabolism: A functional triangle in bioenergetics. Biochim. Biophys. Acta Mol. Cell Res..

[42] Kataoka T, Tschopp J (2004). N-terminal fragment of c-FLIP(L) processed by caspase 8 specifically interacts with TRAF2 and induces activation of the NF-κB signaling pathway. Mol. Cell Biol..

[43] Takahashi Y, Hirose F, Matsukage A, Yamaguchi M (1999). Identification of three conserved regions in the DREF transcription factors from *Drosophila melanogaster* and *Drosophila virilis*. Nucleic Acids Res..

[44] Kondo T (2019). 4-*O*-methylascochlorin inhibits the prolyl hydroxylation of hypoxia-inducible factor-1α, which is attenuated by ascorbate. J. Antibiot..

[45] Matsuda I (2014). The C-terminal domain of the long form of cellular FLICE-inhibitory protein (c-FLIP_L_) inhibits the interaction of the caspase 8 prodomain with the receptor-interacting protein 1 (RIP1) death domain and regulates caspase 8-dependent nuclear factor κB (NF-κB) activation. J. Biol. Chem..

[46] Kadohara K (2009). Caspase-8 mediates mitochondrial release of pro-apoptotic proteins in a manner independent of its proteolytic activity in apoptosis induced by the protein synthesis inhibitor acetoxycycloheximide in human leukemia Jurkat cells. J. Biol. Chem..

