# Supplementary material for: The BCL-2 family protein BCL-RAMBO interacts and cooperates with GRP75 to promote its apoptosis signaling pathway
Source: Sci Rep. 2023 Aug 28;13:14041. doi: 10.1038/s41598-023-41196-0 (PMC10462657; doi:10.1038/s41598-023-41196-0)
Supplement: Supplementary file 1 — Supplementary Figures. [file 41598_2023_41196_MOESM1_ESM.pdf]

Supplementary information

The BCL-2 family protein BCL-RAMBO interacts and cooperates with GRP75 to promote apoptosis signaling pathway

Jinghong Xu [1], Takuya Hashino [1], Reiji Tanaka [1], Koichiro Kawaguchi [1], Hideki Yoshida [1] and Takao Kataoka [1,2,*]

[1] Department of Applied Biology, Kyoto Institute of Technology, Matsugasaki, Sakyo-ku, Kyoto 606-8585, Japan

[2] Biomedical Research Center, Kyoto Institute of Technology, Matsugasaki, Sakyo-ku, Kyoto 606-8585, Japan

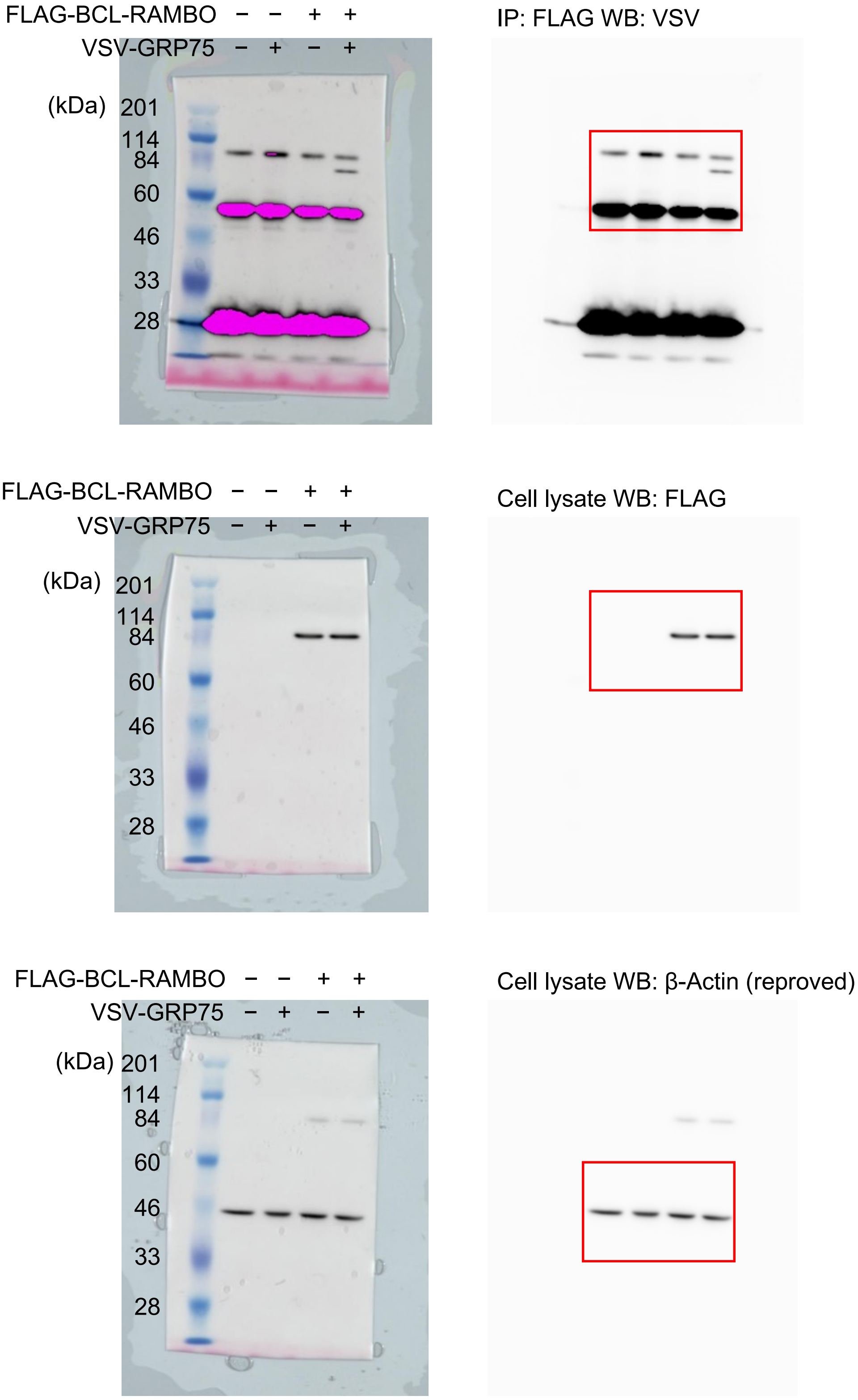

Figure S1 (continued)

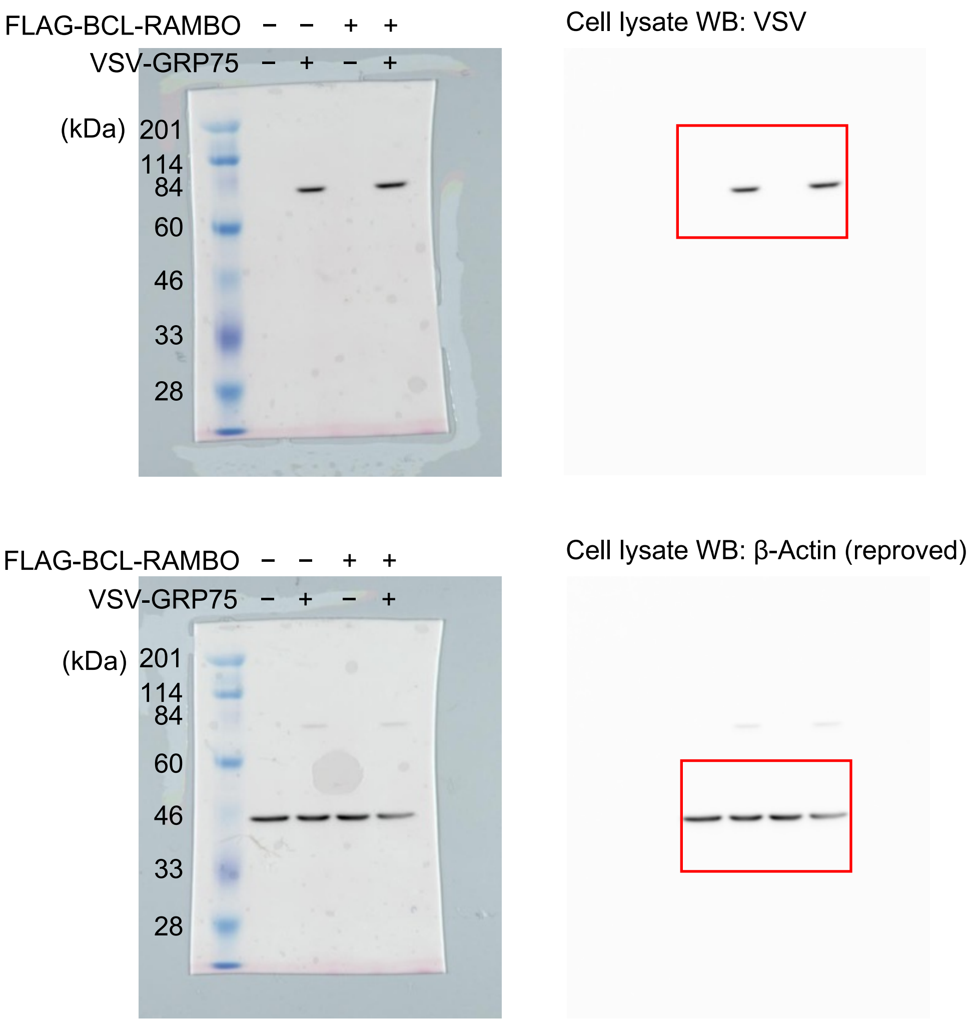

Figure S1. Original blots used in Figure 1a. Blots merged with protein bands and prestained protein markers are shown (left panels). Cropped areas are indicated by red squares in blots with protein bands (right panels).

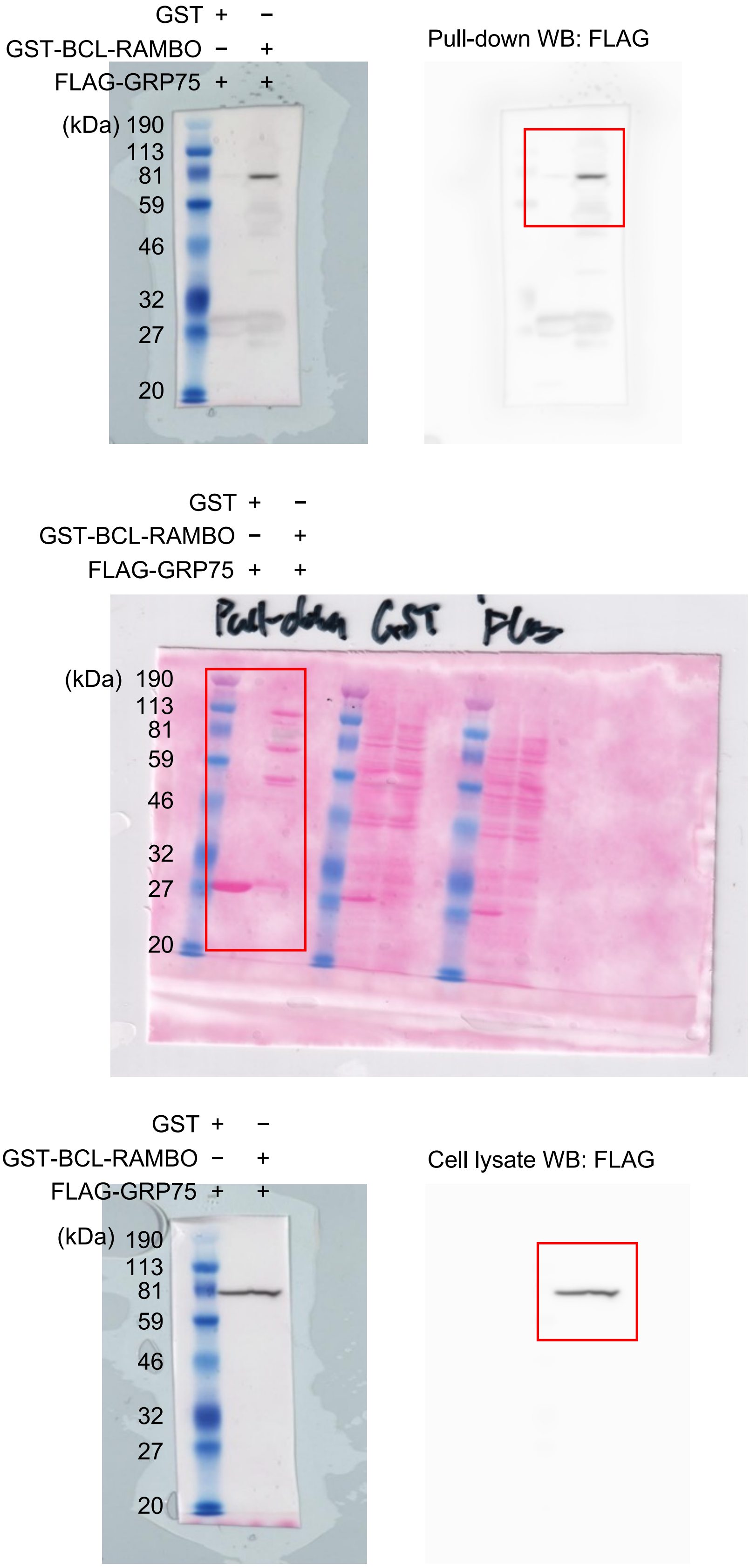

Figure S2 (continued)

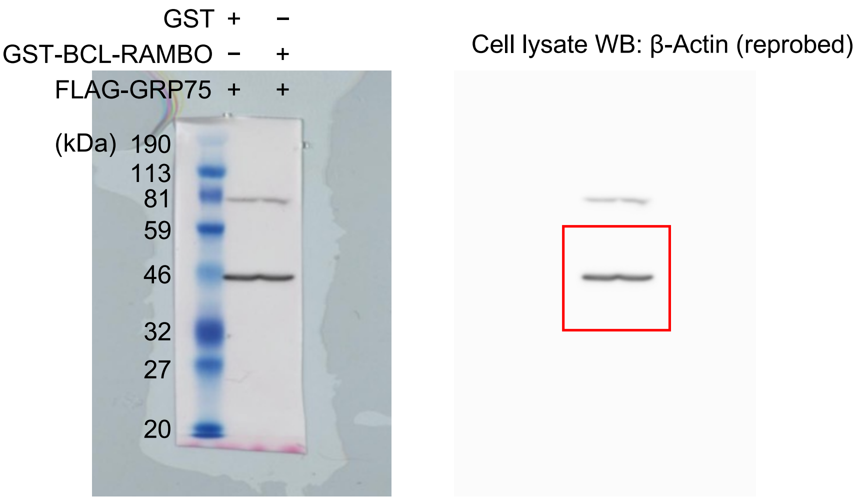

Figure S2. Original blots used in Figure 1b. Blots merged with protein bands and prestained protein markers are shown (left panels). Cropped areas are indicated by red squares in blots with protein bands (right panels). The ponceau S-stained blot and cropped area (red square) are shown.

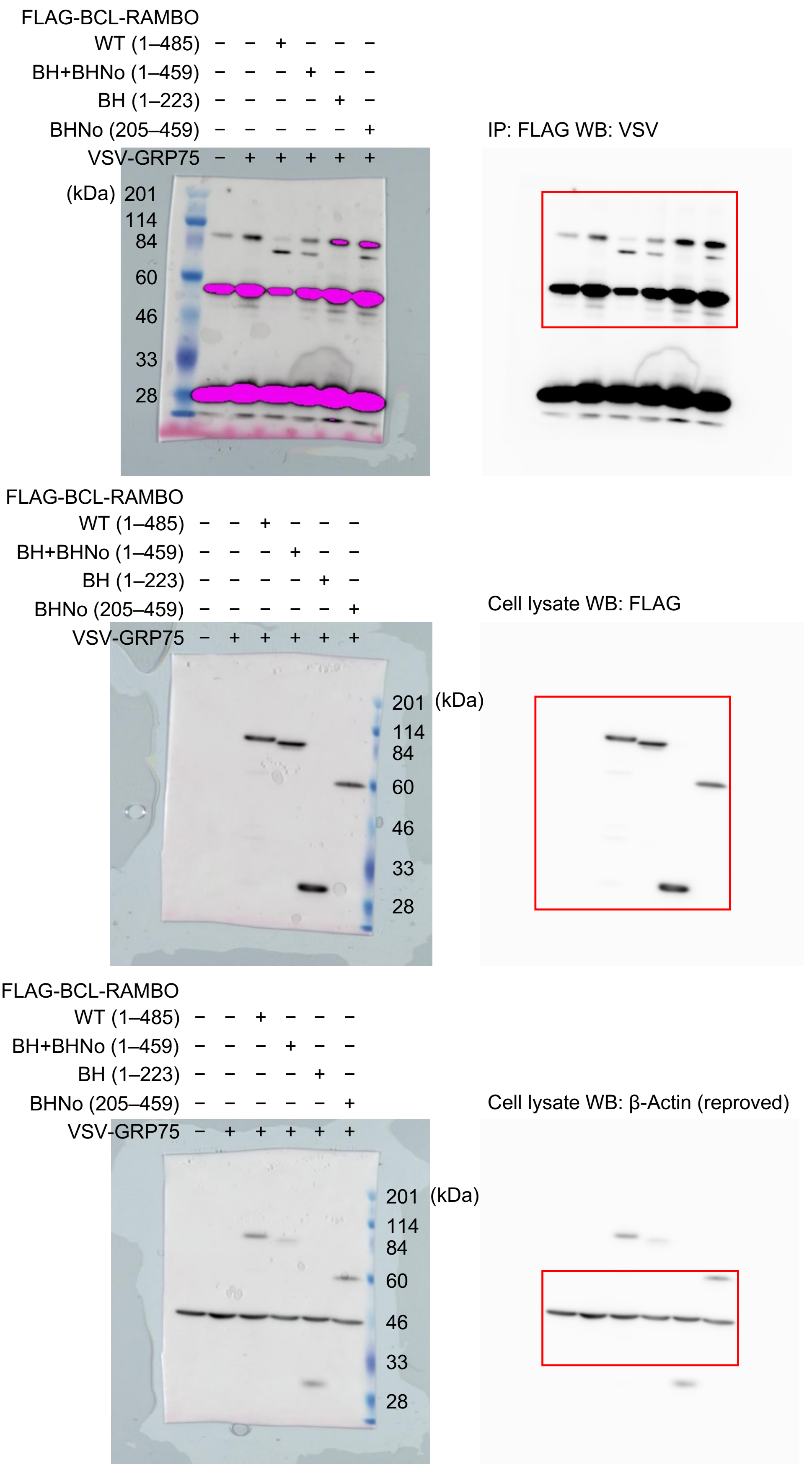

Figure S3 (continued)

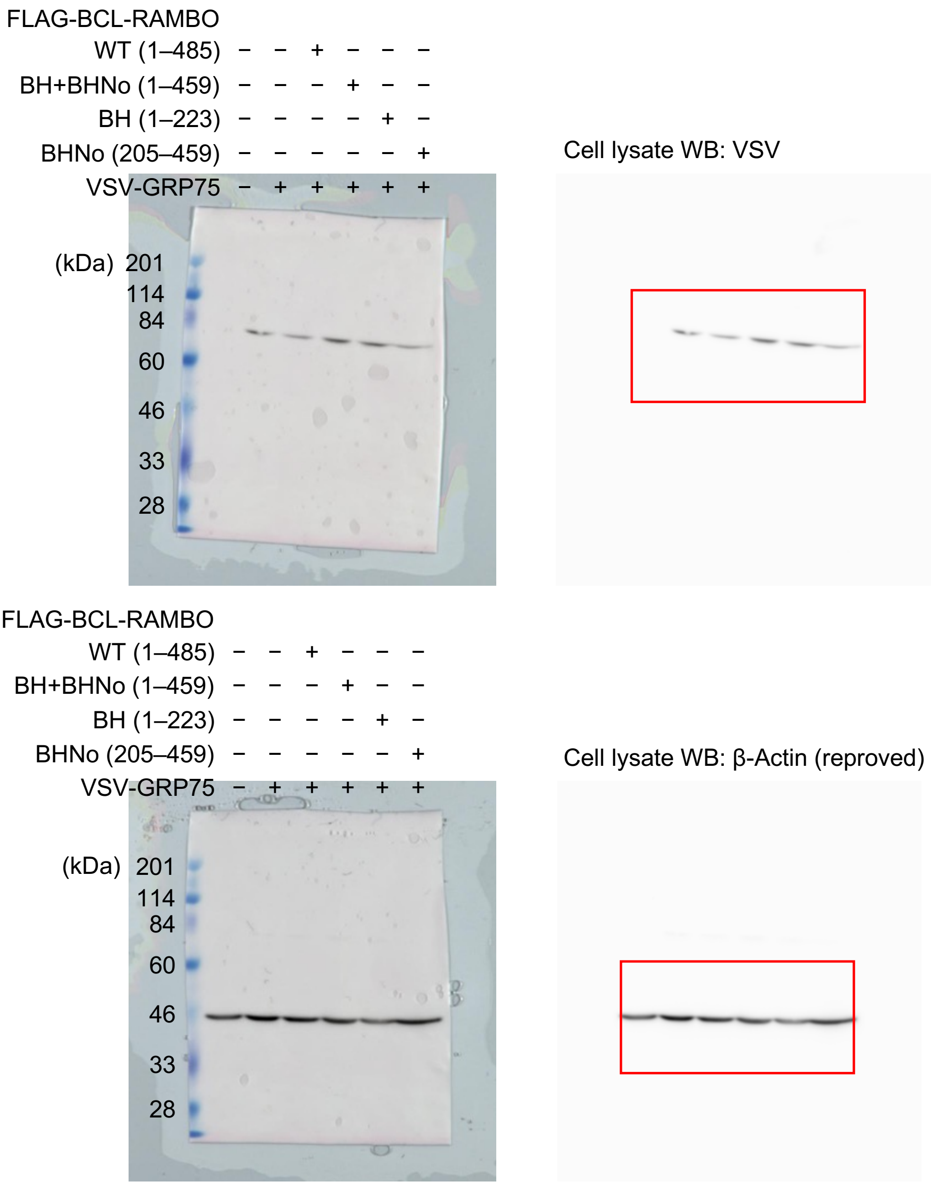

Figure S3. Original blots used in Figure 1d. Blots merged with protein bands and prestained protein markers are shown (left panels). Cropped areas are indicated by red squares in blots with protein bands (right panels).

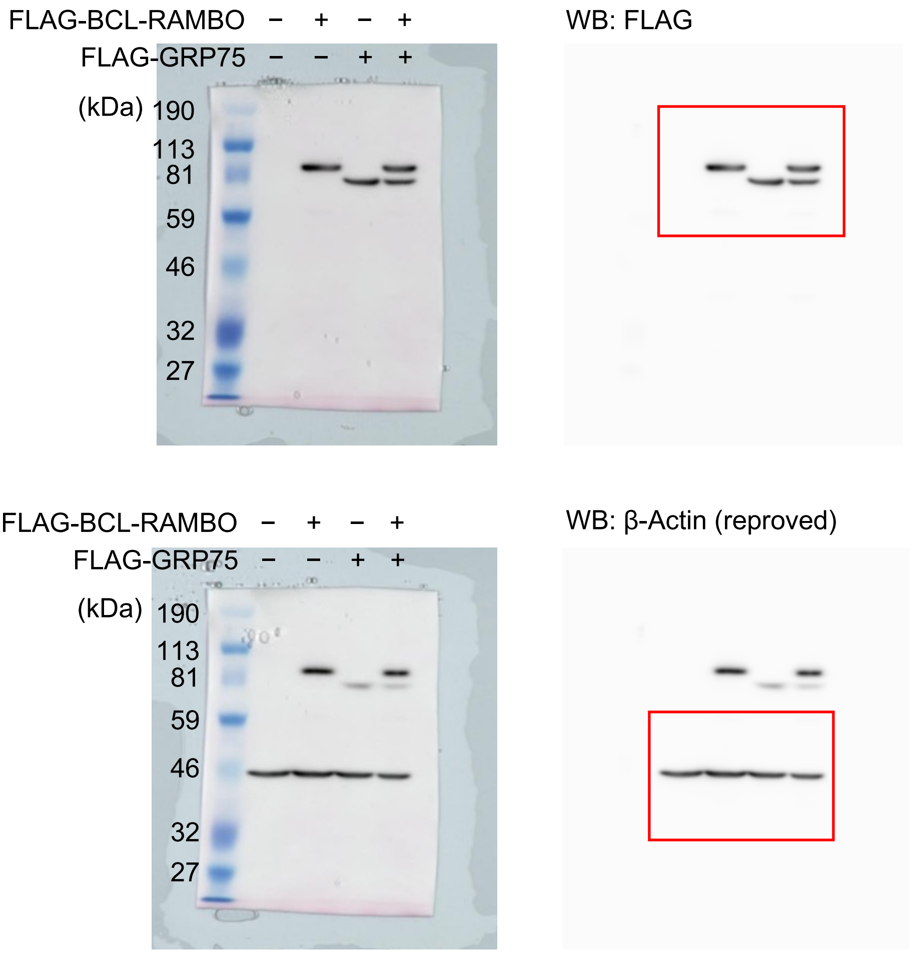

Figure S4. Original blots used in Figure 3a. Blots merged with protein bands and prestained protein markers are shown (left panels). Cropped areas are indicated by red squares in blots with protein bands (right panels).

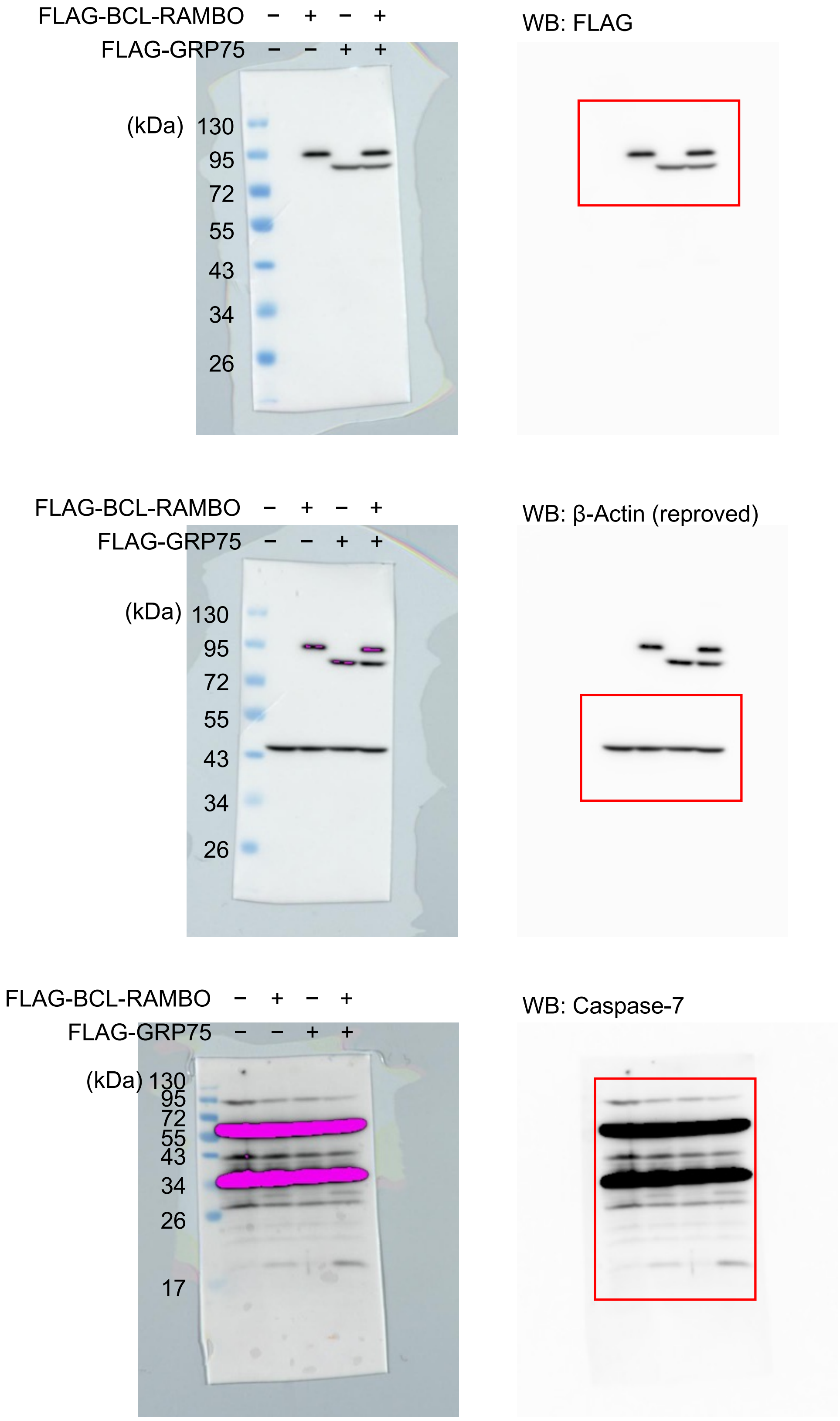

Figure S5 (continued)

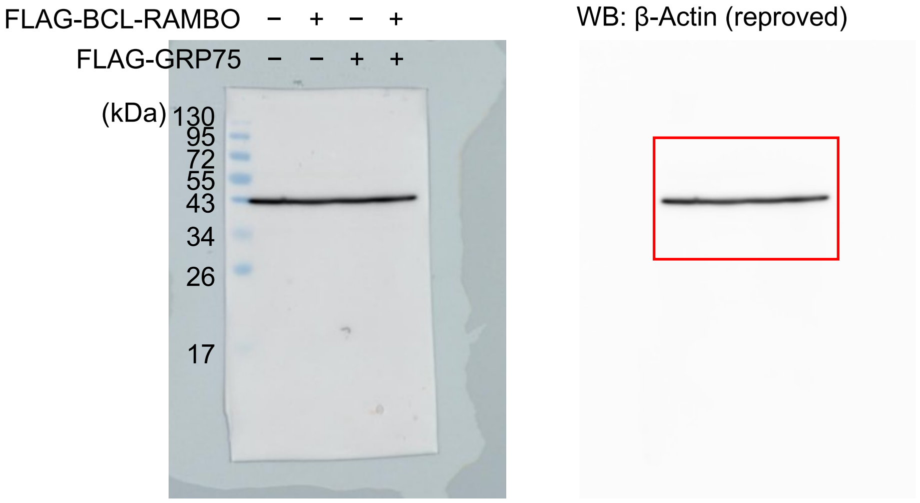

Figure S5. Original blots used in Figure 3c. Blots merged with protein bands and prestained protein markers are shown (left panels). Cropped areas are indicated by red squares in blots with protein bands (right panels).

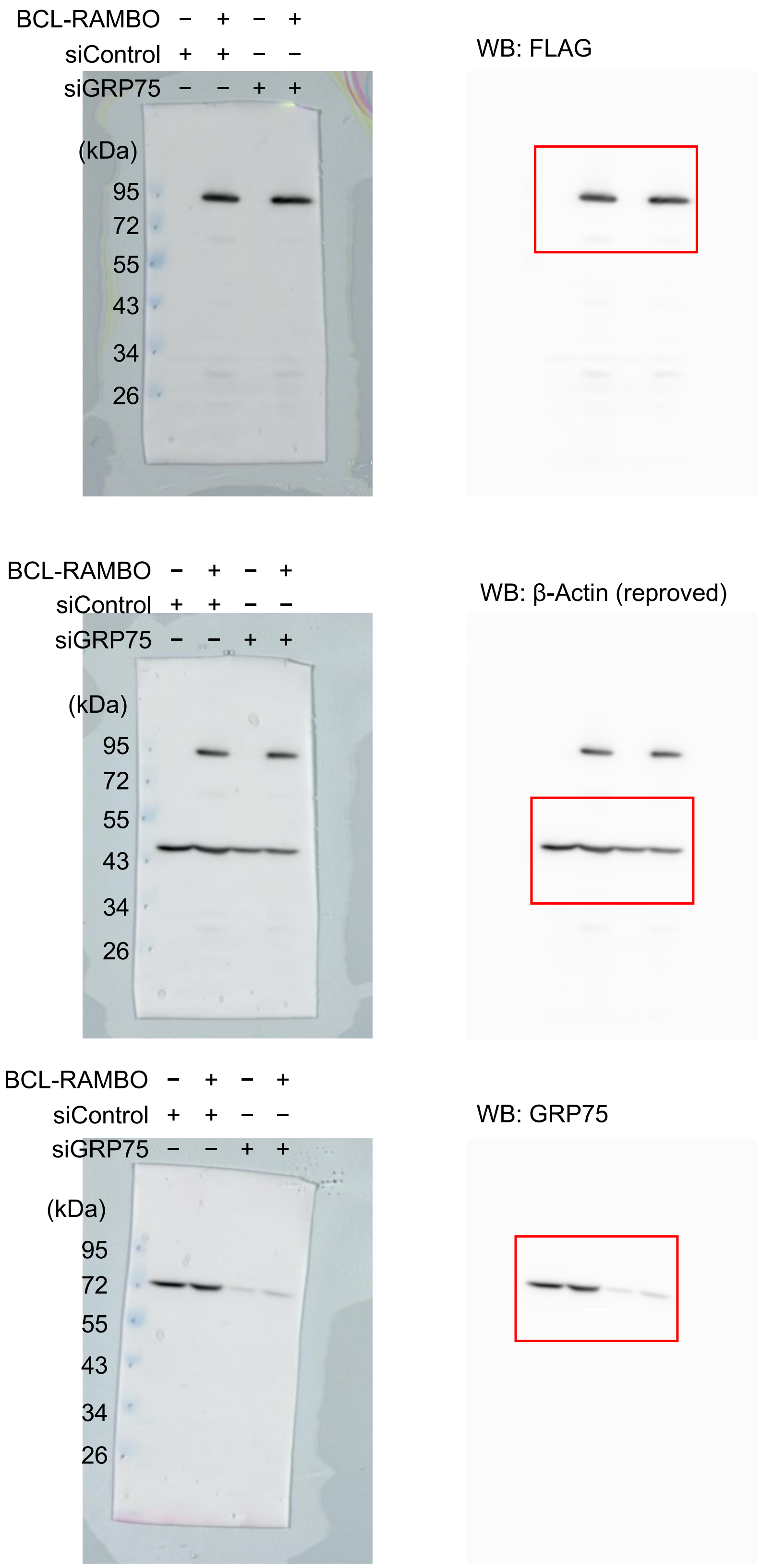

Figure S6 (continued)

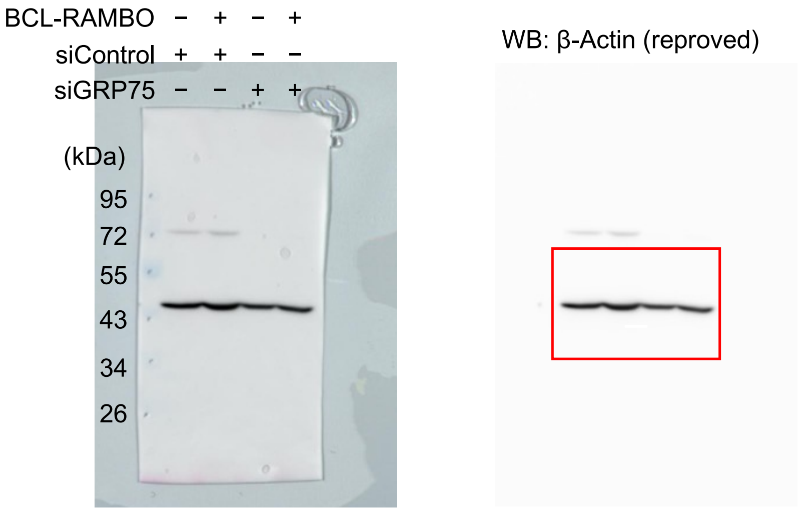

Figure S6. Original blots used in Figure 3e. Blots merged with protein bands and prestained protein markers are shown (left panels). Cropped areas are indicated by red squares in blots with protein bands (right panels).

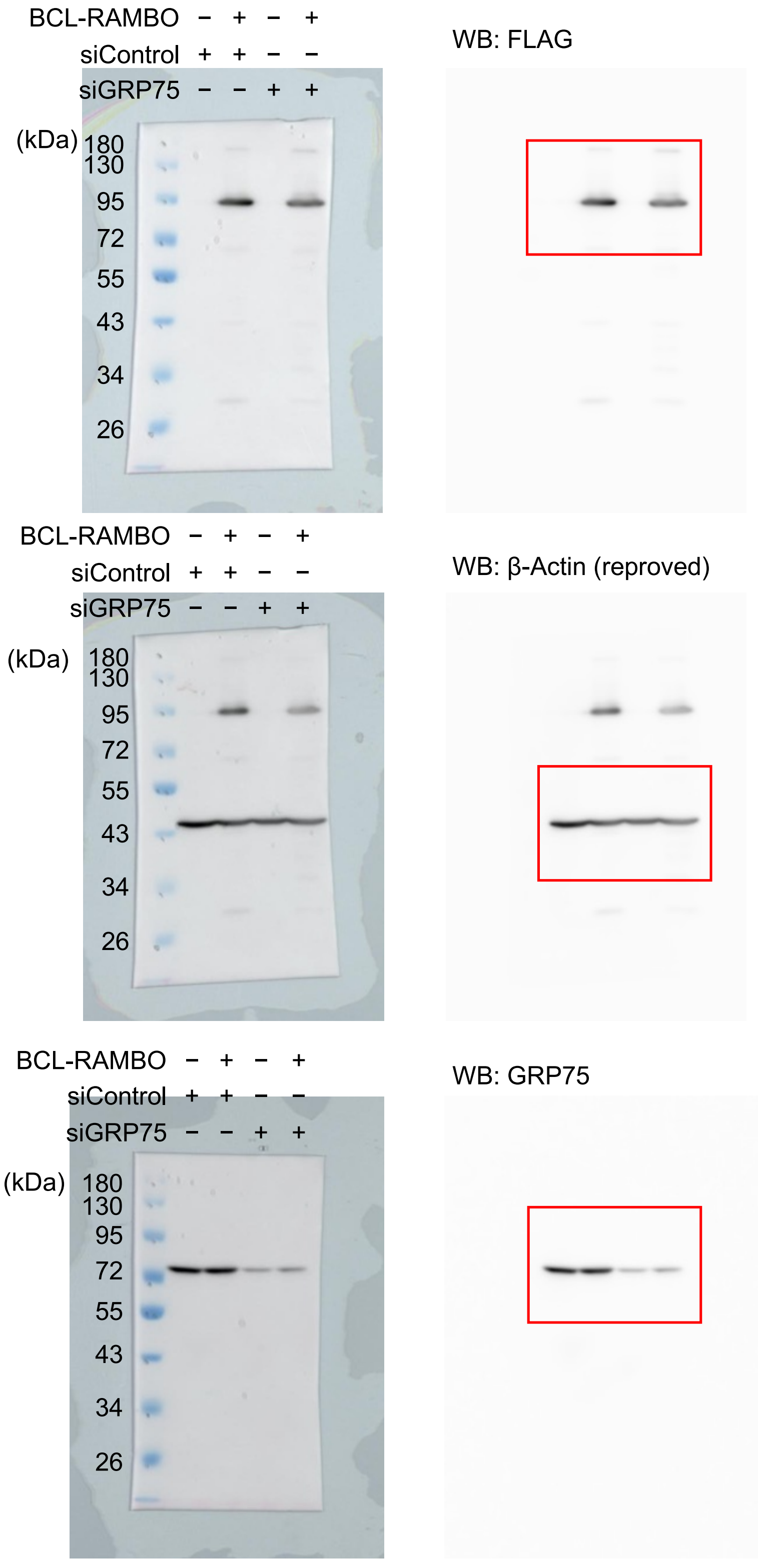

Figure S7 (continued)

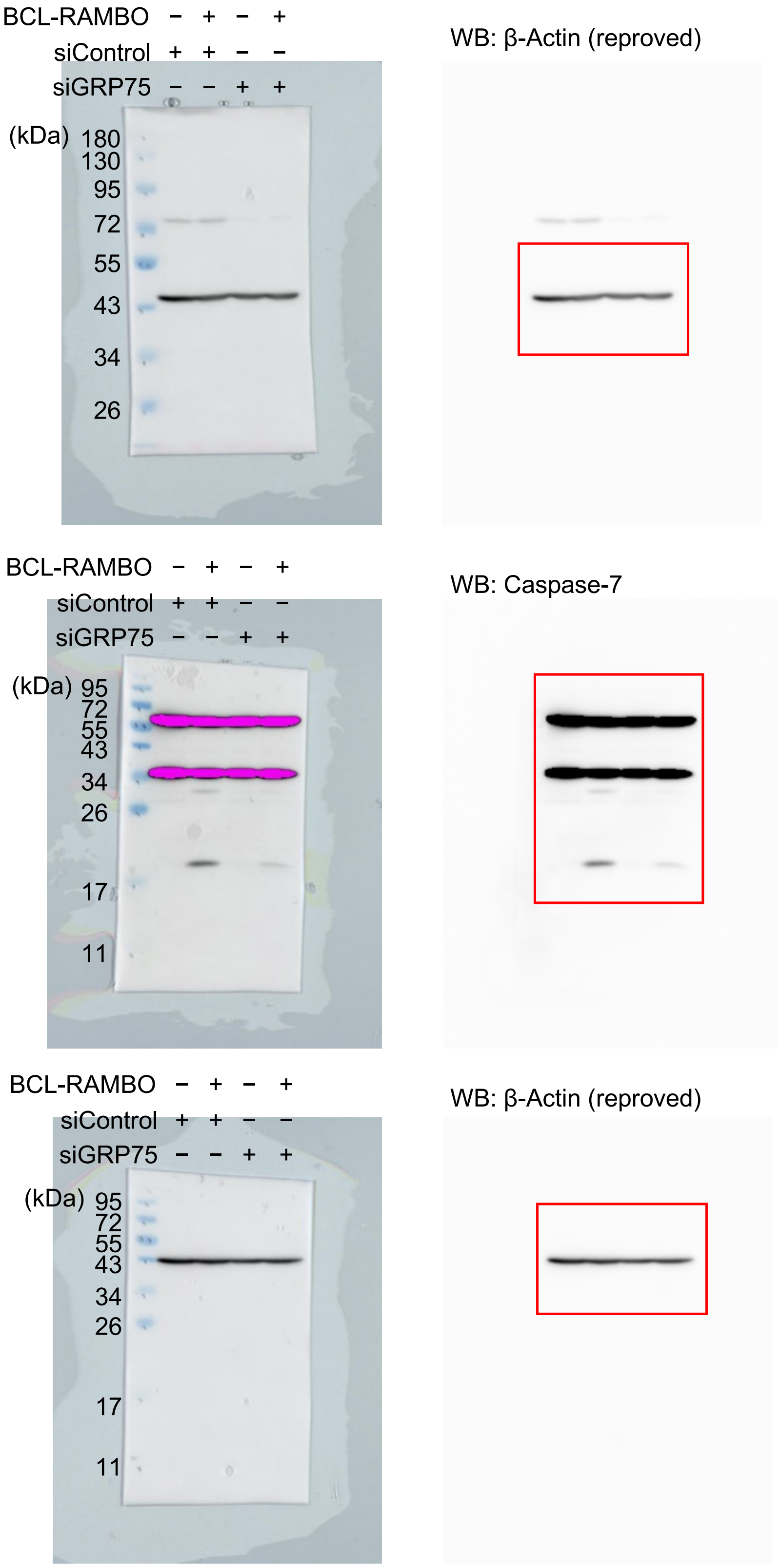

Figure S7. Original blots used in Figure 3h. Blots merged with protein bands and prestained protein markers are shown (left panels). Cropped areas are indicated by red squares in blots with protein bands (right panels).

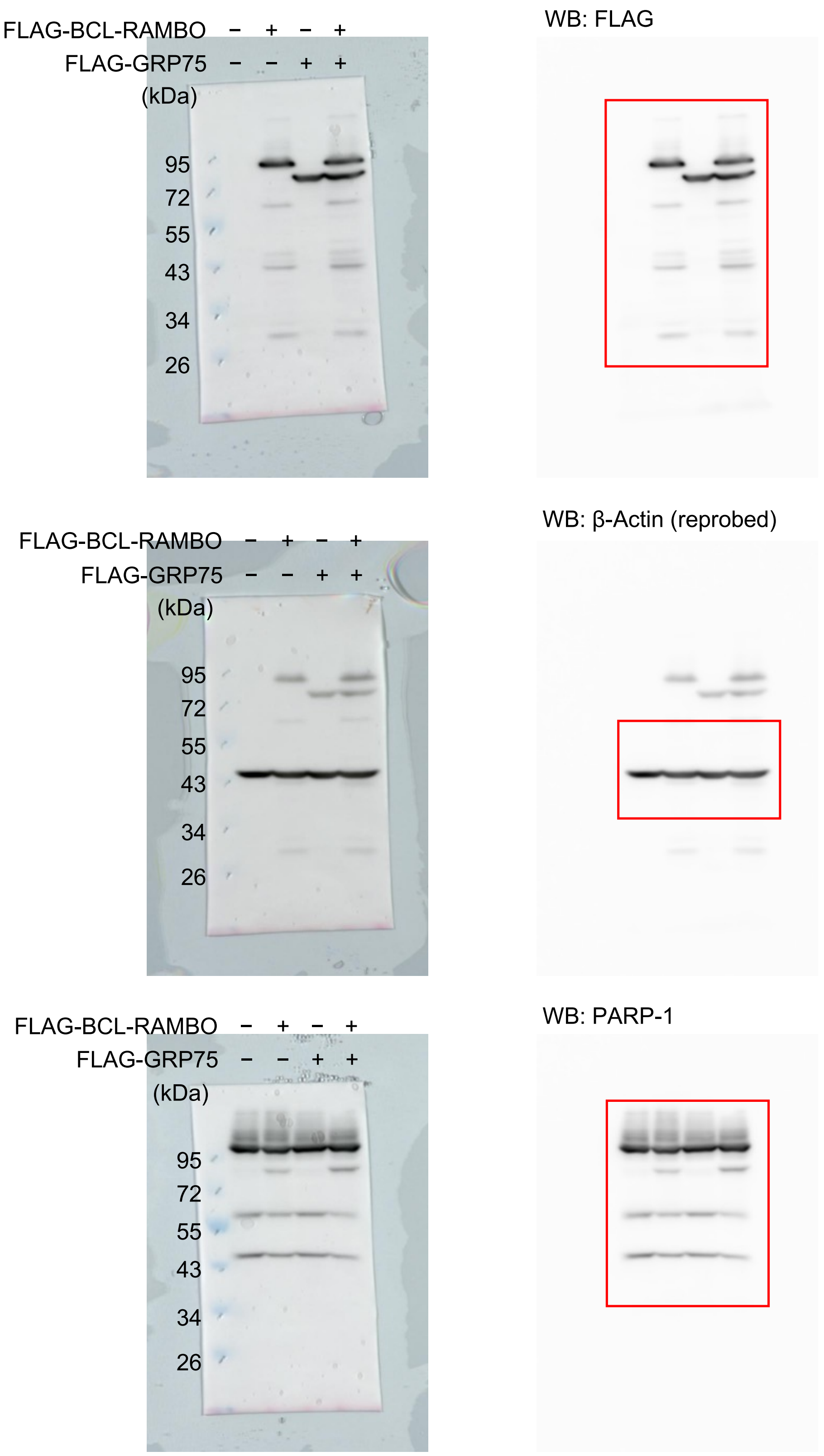

Figure S8 (continued)

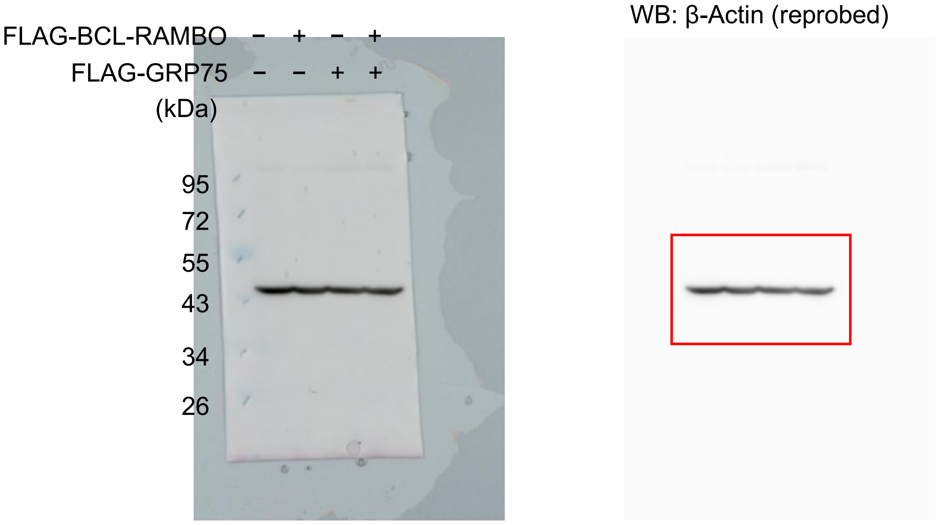

Figure S8. Original blots used in Figure 4a. Blots merged with protein bands and prestained protein markers are shown (left panels). Cropped areas are indicated by red squares in blots with protein bands (right panels).

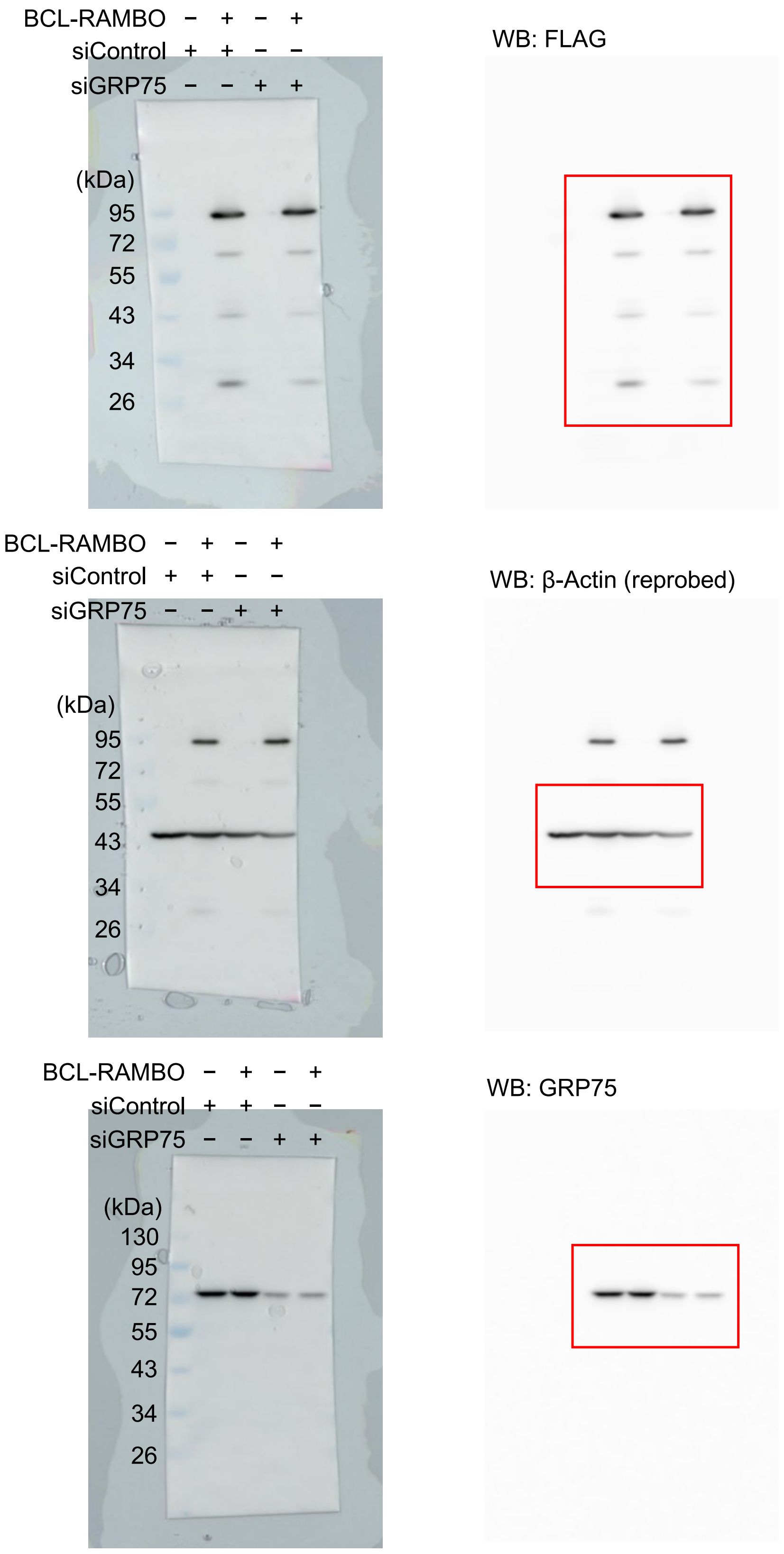

Figure S9 (continued)

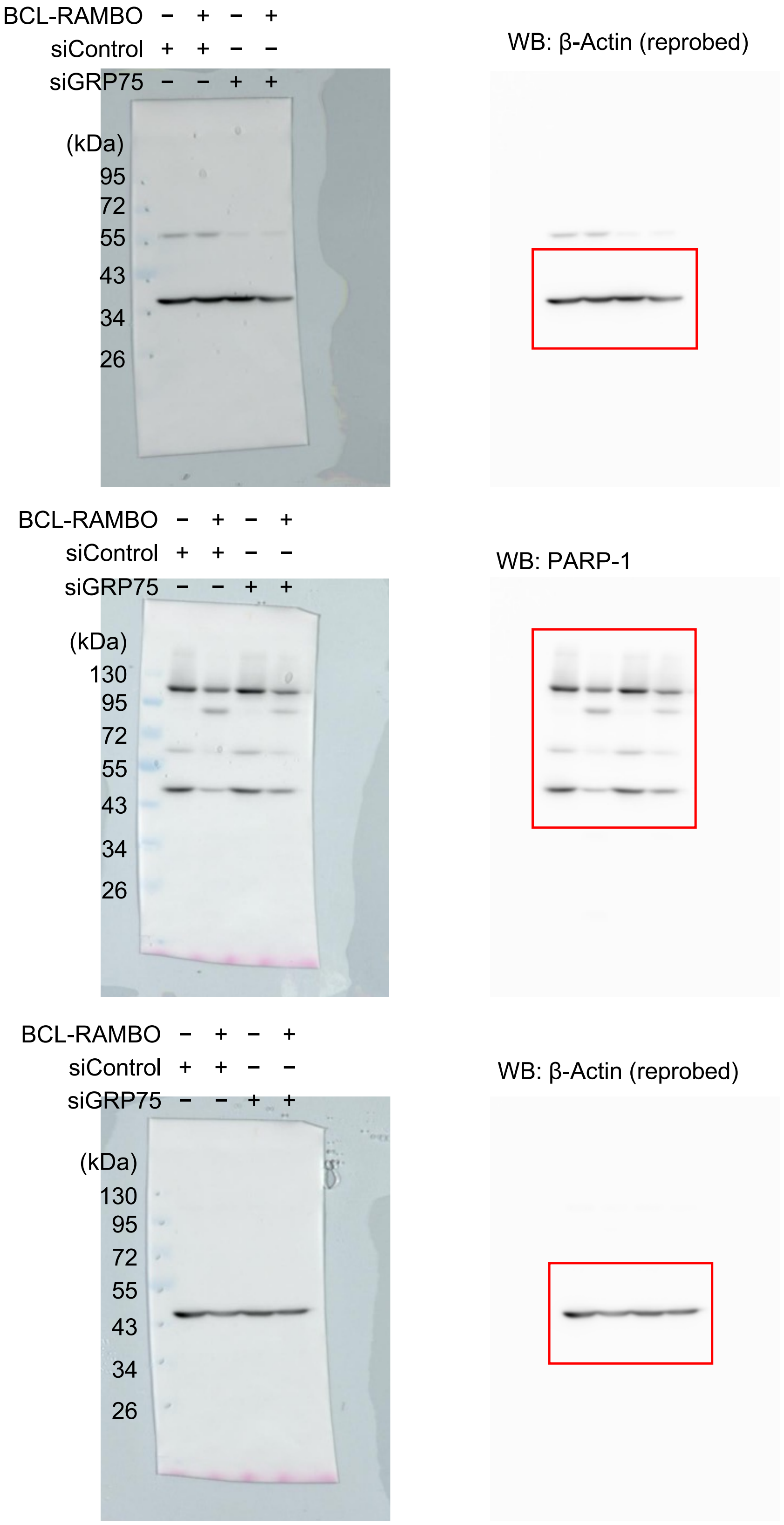

Figure S9. Original blots used in Figure 4c. Blots merged with protein bands and prestained protein markers are shown (left panels). Cropped areas are indicated by red squares in blots with protein bands (right panels).

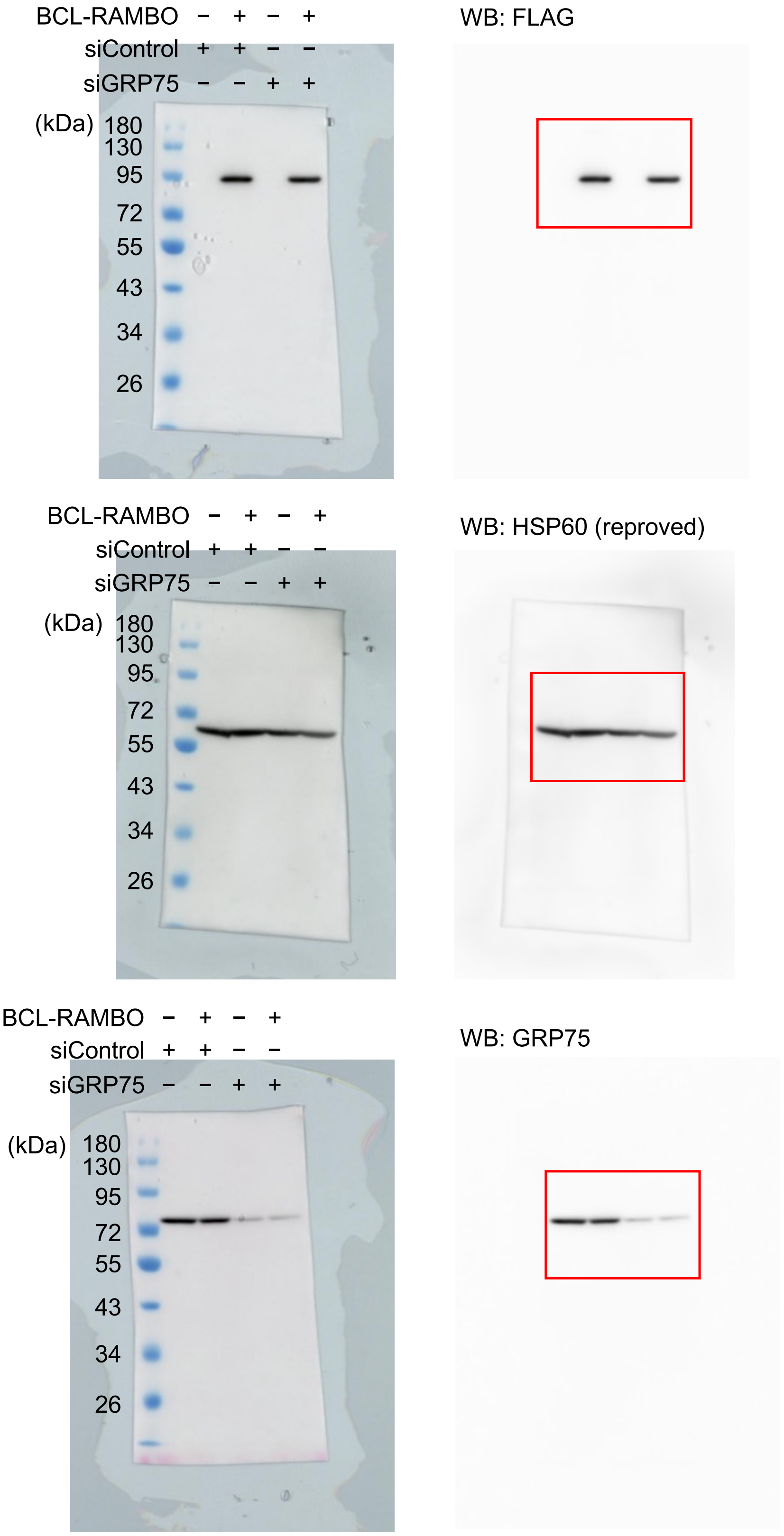

Figure S10 (continued)

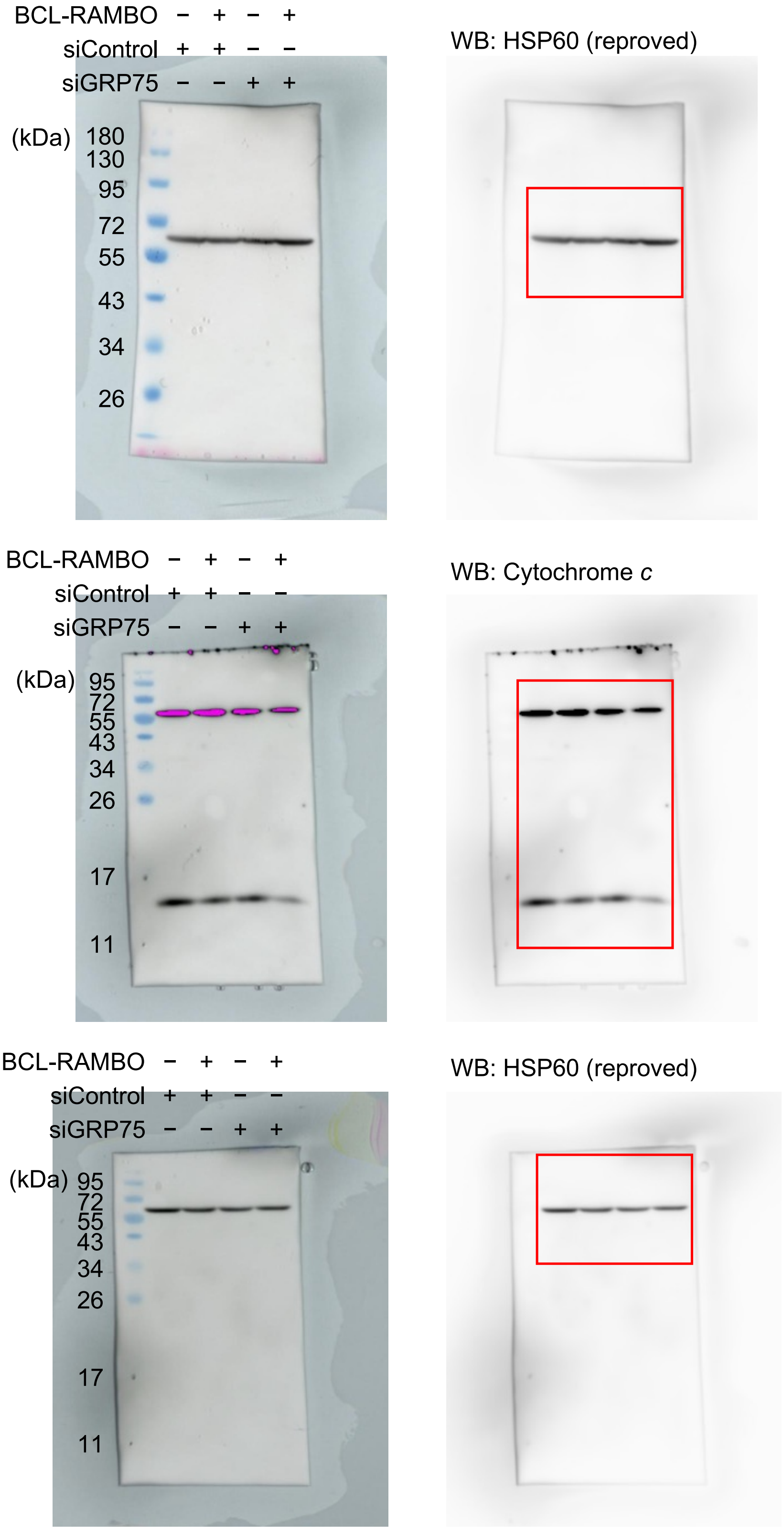

Figure S10. Original blots used in Figure 5a. Blots merged with protein bands and prestained protein markers are shown (left panels). Cropped areas are indicated by red squares in blots with protein bands (right panels).

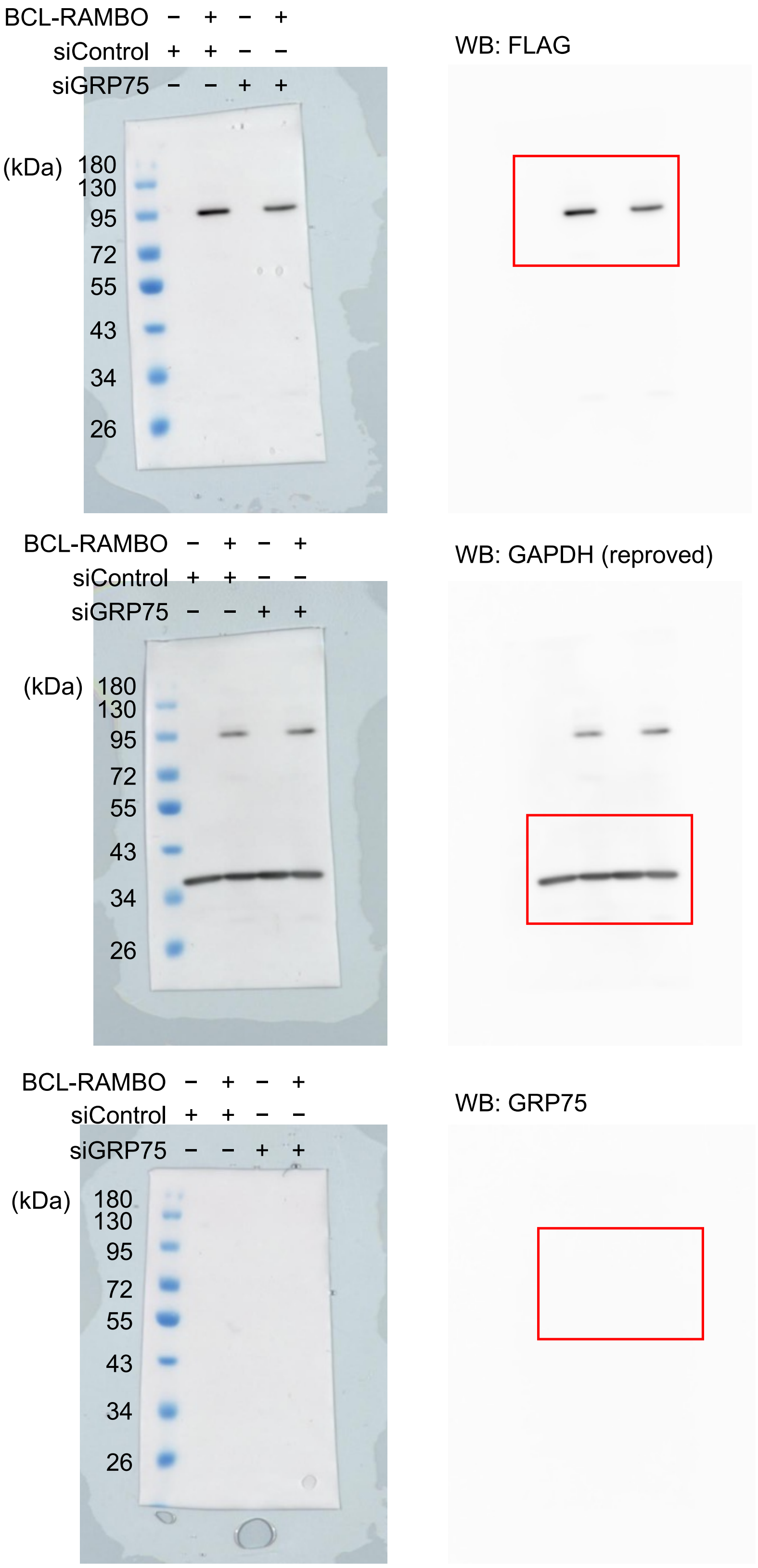

Figure S11 (continued)

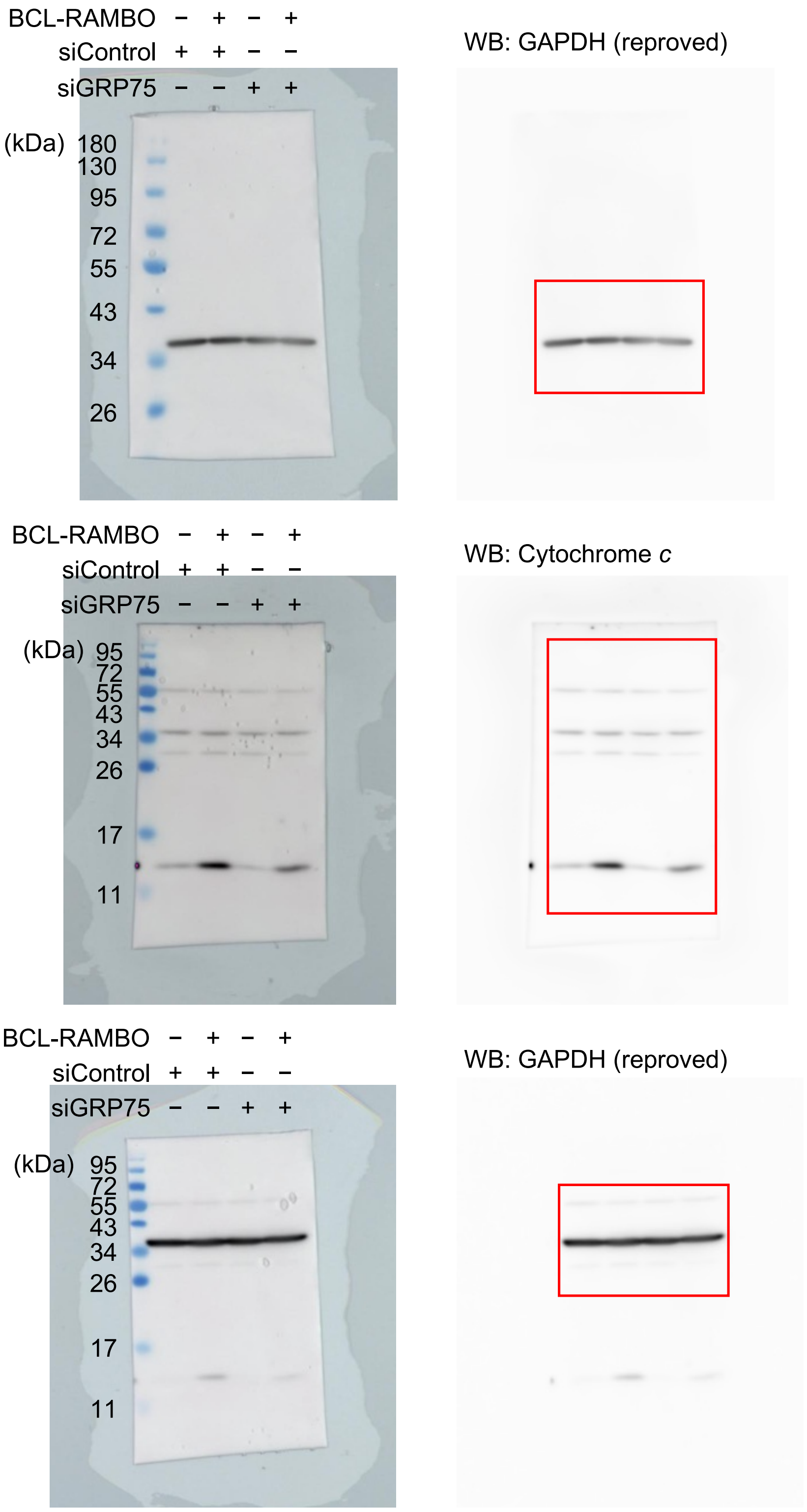

Figure S11. Original blots used in Figure 5b. Blots merged with protein bands and prestained protein markers are shown (left panels). Cropped areas are indicated by red squares in blots with protein bands (right panels).

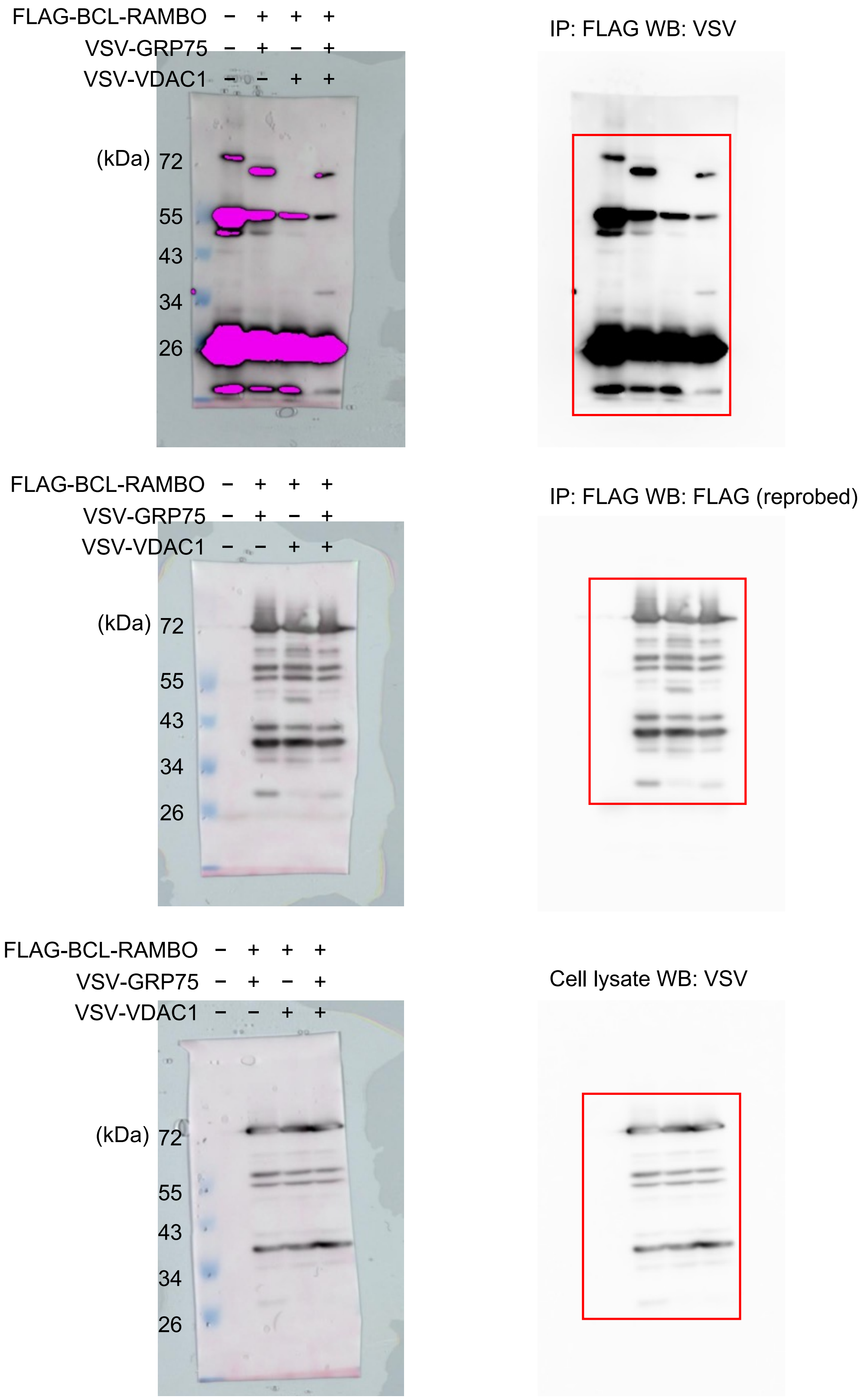

Figure S12 (continued)

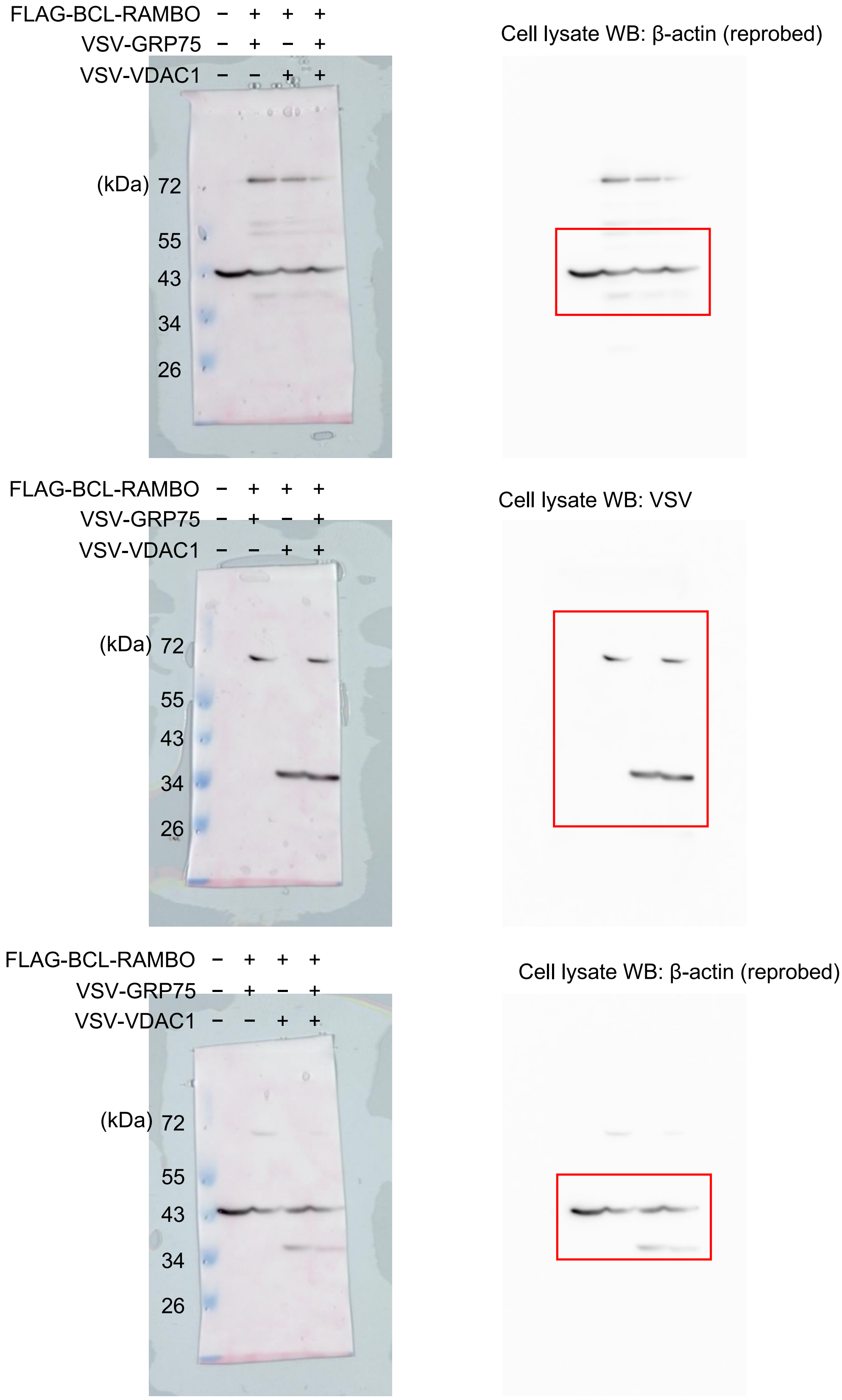

Figure S12. Original blots used in Figure 6. Blots merged with protein bands and prestained protein markers are shown (left panels). Cropped areas are indicated by red squares in blots with protein bands (right panels).
